# RNase H1 promotes replication fork progression through oppositely transcribed regions of *Drosophila* mitochondrial DNA

**DOI:** 10.1074/jbc.RA118.007015

**Published:** 2019-01-11

**Authors:** Jose M. González de Cózar, Mike Gerards, Eveliina Teeri, Jack George, Eric Dufour, Howard T. Jacobs, Priit Jõers

**Affiliations:** From the ‡ Faculty of Medicine and Health Technology and Tampere University Hospital, FI-33014 Tampere University, Finland,; § Institute of Biotechnology, FI-00014 University of Helsinki, Finland, and; ¶ Institute of Molecular and Cell Biology, University of Tartu, Riia 23, 51010 Tartu, Estonia

**Keywords:** mitochondria, DNA replication, RNA, genomic instability, ribonuclease, heteroduplex, locomotor dysfunction, mtDNA, replication fork, RNase H, genome stability, insect rnh1

## Abstract

Mitochondrial DNA (mtDNA) replication uses a simple core machinery similar to those of bacterial viruses and plasmids, but its components are challenging to unravel. Here, we found that, as in mammals, the single *Drosophila* gene for RNase H1 (*rnh1*) has alternative translational start sites, resulting in two polypeptides, targeted to either mitochondria or the nucleus. RNAi-mediated *rnh1* knockdown did not influence growth or viability of S2 cells, but compromised mtDNA integrity and copy number. *rnh1* knockdown in intact flies also produced a phenotype of impaired mitochondrial function, characterized by respiratory chain deficiency, locomotor dysfunction, and decreased lifespan. Its overexpression in S2 cells resulted in cell lethality after 5–9 days, attributable to the nuclearly localized isoform. *rnh1* knockdown and overexpression produced opposite effects on mtDNA replication intermediates. The most pronounced effects were seen in genome regions beyond the major replication pauses where the replication fork needs to progress through a gene cluster that is transcribed in the opposite direction. RNase H1 deficiency led to an accumulation of replication intermediates in these zones, abundant mtDNA molecules joined by four-way junctions, and species consistent with fork regression from the origin. These findings indicate replication stalling due to the presence of unprocessed RNA/DNA heteroduplexes, potentially leading to the degradation of collapsed forks or to replication restart by a mechanism involving strand invasion. Both mitochondrial RNA and DNA syntheses were affected by *rnh1* knockdown, suggesting that RNase H1 also plays a role in integrating or coregulating these processes in *Drosophila* mitochondria.

## Introduction

Mitochondrial DNA (mtDNA)^3^ replication utilizes a relatively simple core machinery, akin to those of bacterial viruses and plasmids. It proceeds via mechanisms that reflect this but in other respects exhibits unique features (1, 2). The mitochondrial genome of metazoans has been the most intensively studied (1), in part because of its relevance to human pathology (3). Its replication depends on a small set of well-studied proteins, including a dedicated DNA polymerase (Pol γ), RNA polymerase, and DNA helicase, all related to those of the T-odd bacteriophages (4), as well as other proteins involved in DNA compaction and transcription (2). The latter notably includes mitochondrial transcription factor A (TFAM) (5, 6), which functions in both processes (7–10), several other transcription factors (11–13), the Pol γ accessory subunit (14–16), and the mitochondrial homologue of bacterial single-strand DNA-binding protein, mtSSB (17, 18). The full set of proteins required for mtDNA replication *in vivo* and their precise functions have not yet been defined.

The study of mtDNA replication intermediates by EM, two-dimensional neutral agarose gel electrophoresis (2DNAGE), and other methods (19–22), plus the use of cell-free replication systems *in vitro* (23, 24) and *in organello* (25, 26), has indicated a multiplicity of roles in the replication process for RNA and for transient RNA/DNA heteroduplexes. It remains unclear whether the observed heteroduplexes reflect aspects of a single mtDNA replication process or several such mechanisms operating in parallel. Furthermore, their significance, if any, for RNA synthesis is unknown.

In seeking to identify all of the components of the mtDNA replication machinery, whether by genetic or biochemical means, an inherent problem arises if a relevant gene product also functions in the cell nucleus. One example that has attracted our attention is RNase H1 (27), mutants of which are associated with human disease (28, 29) and which in the mouse is required to maintain mtDNA both *in vivo* and in cultured cells (30, 31). RNase H1 cleaves the RNA strand of RNA/DNA hybrids, requiring as substrate at least four consecutive ribonucleotides (32). The mammalian gene for RNase H1 encodes two variant polypeptides, which have been shown to be targeted, respectively, to the nucleus or to mitochondria, by virtue of alternate translation starts that define distinct N-terminal peptide sequences (27), with tight regulation by a short upstream ORF.

In the mouse, deletion of the *Rnaseh1* gene results in embryonic lethality at day 8.5 (30), a phenotype similar to that seen in mice lacking other essential components of the mtDNA replication apparatus such as Pol γ (33) or TFAM (5). However, this does not constitute formal proof that the lethality is due to effects on mtDNA alone, and the exact role of the enzyme in each compartment has not yet been fully defined. Moreover, the physiological function of the enzyme, if any, in the adult could not be ascertained.

In mammalian mitochondria, RNase H1 has been inferred to remove RNA fragments believed to represent unprocessed primers, a process required for the completion of mtDNA replication (31), and to facilitate the separation of daughter copies (29) as well as some aspects of rRNA processing (34). In mammals, the RITOLS mode of mtDNA replication (transient RNA incorporation throughout the lagging strand; Refs. 20 and 21) also has an intrinsic requirement for the processing of intermediates containing tracts of RNA/DNA heteroduplex. In the nucleus, RNase H1 is required to eliminate persistent heteroduplexes that impair transcription (35) or impede fork progression during DNA replication (36), leading to genome instability. It has also been suggested to play roles in other processes, such as telomere maintenance (37), retroelement surveillance (19), and DNA repair (38), as well as facilitating somatic hypermutation in the immunoglobulin locus (39).

The *Drosophila* mitochondrial genome is similar to that of vertebrates, except for two major inversions that balance the coding capacity of the two strands. The extended noncoding region (NCR) in which the replication origin is embedded (40, 41) is also unusually A + T–rich (∼95%) and contains several long repeat elements, clustered in two blocks. *Drosophila* mtDNA follows the unidirectional, θ-type replication model also seen in mammals with some minor, but intriguing differences. The initial portion of the genome appears to be replicated by strand displacement, with delayed lagging-strand synthesis (40), as proposed for mammalian mtDNA (42). However, beyond a site near the start of the coding region, replication switches to a strand-coupled mode (40). In contrast to mammals, where this can entail the incorporation of extended tracts of lagging-strand RNA, only short heteroduplexes are formed transiently at the replication fork (13) during elongation.

*Drosophila* is a convenient model organism in which to study mtDNA maintenance because of the plethora of genetic tools available as well as the fact that the most commonly used *Drosophila* cell line (Schneider S2 cells) is able to survive for long periods essentially without mtDNA-encoded functions. S2 cells are also susceptible to highly effective and specific RNAi brought about by long double-stranded RNAs (dsRNAs). In an RNAi screen for *Drosophila* genes required for the maintenance of mtDNA nucleoids in S2 cells (43), we identified those encoding the previously identified “core set” of replication proteins, whose knockdown by RNAi resulted in depletion of mtDNA over 5 days in culture by at least 50%. In contrast, knockdown of RNase H1 (encoded by the *rnh1* gene) did not result in significant mtDNA depletion over this period, nor did it cause cell death, although it did abolish the topology-dependent staining of mtDNA nucleoids by the dye Pico Green (43). Nevertheless, an earlier study identified *rnh1* as an essential gene for the completion of development in the fly (44).

To unravel the potential roles of *rnh1* in *Drosophila* and in particular its relevance to mtDNA metabolism and to the physiology of the adult, we first created reporter constructs to test its subcellular localization. After confirming that it is targeted to mitochondria as well as to the nucleus, we examined the effects on cell and organismal phenotype and on mtDNA of its knockdown or overexpression. Our findings imply that RNase H1 is required to prevent or resolve conflicts between opposed mtDNA transcription and replication in addition to a role at the origin analogous to that inferred previously in mammalian mtDNA. We infer that RNase H1 is essential to ensure faithful genome maintenance and expression by removing heteroduplex from critical regions. Furthermore, these mitochondrial functions of the enzyme are inferred to be essential to the maintenance of locomotor competence and lifespan in the adult fly.

## Results

### RNaseH1 is targeted to both mitochondria and the nucleus in Drosophila

**Figure 1.**
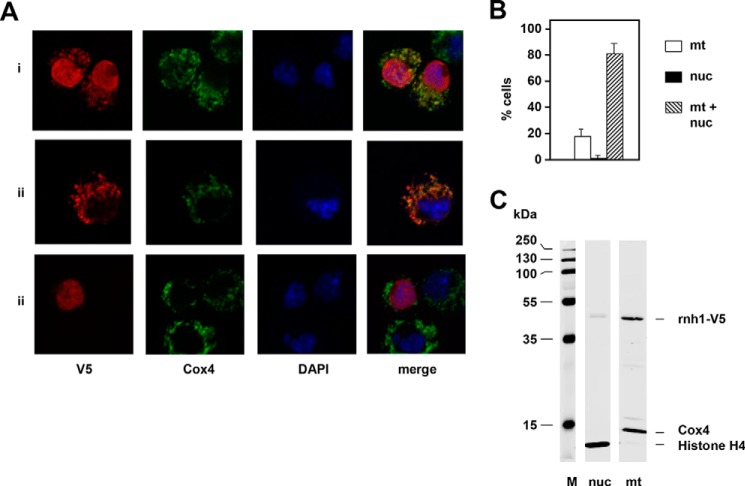
**Subcellular localization of epitope-tagged RNase H1.** *A*, immunocytochemistry of cells transiently transfected with RNase H1-V5, probed for the V5 epitope tag (*red*), Cox4 (*green*), and DAPI (*blue*), showing examples of the three types of intracellular distribution of V5-tagged RNase H1: nucleus and mitochondria (*i*), mitochondria only (*ii*), and nucleus only (*iii*). *B*, subcellular distribution of RNase H1-V5 in 100 transfected cells as indicated (mean of three experiments, *error bars* denote S.D.). *C*, Western blots of subcellular fractions from cells transfected with RNase H1-V5, highly enriched for nuclei (*nuc*) or mitochondria (*mt*) as indicated, probed simultaneously for V5 and for the markers indicated. *M*, molecular mass markers.

**Figure 2.**
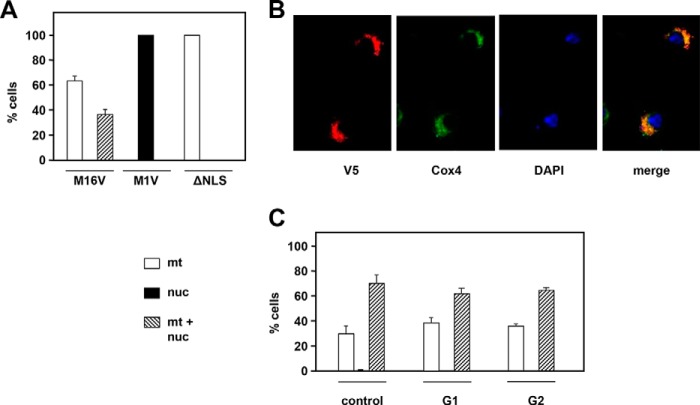
**Subcellular targeting of RNase H1 variants.** *A*, intracellular localization of RNase H1-V5 variants in cultures of stably transfected cells exemplified in *B*. M1V and M16V, N-terminal methionine variants (see Fig. S2*A*); ΔNLS, with the putative nuclear localization signal deleted (see Fig. S2*C*). *C*, intracellular localization of RNase H1-V5 in cells synchronized in G1 and G2 (see FACS profiles in Fig. S2*E*). All plotted values are means of three experiments. *Error bars* denote S.D. *nuc*, nuclei; *mt*, mitochondria.

Having verified that only one mRNA isoform of *rnh1* mRNA is detectable in S2 cells or flies (Fig. S1*B*), we proceeded to investigate the subcellular localization of the encoded protein by a C-terminal epitope-tagging approach (Fig. 1, *A* and *B*). In transient transfections, most cells showed staining in both the nucleus and mitochondria (colocalized with cytochrome *c* oxidase subunit 4 (Cox4)), with a minority showing exclusively mitochondrial localization and a small number (fewer than 2%) exclusively nuclear. Dual targeting was confirmed by Western blotting of subcellular fractions highly enriched for either nuclear or mitochondrial markers (Fig. 1*C*). The N-terminal region of the *rnh1* mRNA contains three AUGs, arranged similarly as in mammals (Fig. S2*A*). The first, shown in purple in Fig. S2*A*, defines a short upstream ORF unrelated to RNase H1 protein, which is considered a signature of translational regulation (45). The remaining two AUGs are in-frame with each other and potentially specify variant polypeptides that differ in their likelihood of being targeted to mitochondria according to three commonly used prediction programs (Fig. S2*B*). To determine whether either of these two AUGs was able to be used as a start codon and how this influenced subcellular targeting, we studied two further epitope-tagged variants with either of the methionine codons at positions 1 and 16 altered to a valine codon. The M1V variant was exclusively localized to nuclei (Fig. 2*A* and S2*D*), whereas the M16V variant was mainly in mitochondria (Fig. 2*A*, S2*D*) with approximately one-third of the cells showing a lower, but still detectable signal in the nucleus. Deletion of the putative nuclear localization signal (Fig. S2*C*, ΔNLS) resulted in purely mitochondrial targeting (Fig. 2, *A* and *B*) despite the presence of both putative start codons. Western blotting using the intact or M16V construct detected two polypeptides, whereas M1V and ΔNLS gave only one (Fig. S2*E*). Cell synchronization in G_1_ by treatment with hydroxyurea for 24 h (Fig. S2*F*) or in G_2_ by treatment with ponasterone A (Fig. S2*F*) revealed no significant differences in the subcellular localization of epitope-tagged RNase H1 compared with unsynchronized growing cells (Fig. 2*C*).

### RNase H1 deficiency produces mitochondrial defects

**Figure 3.**
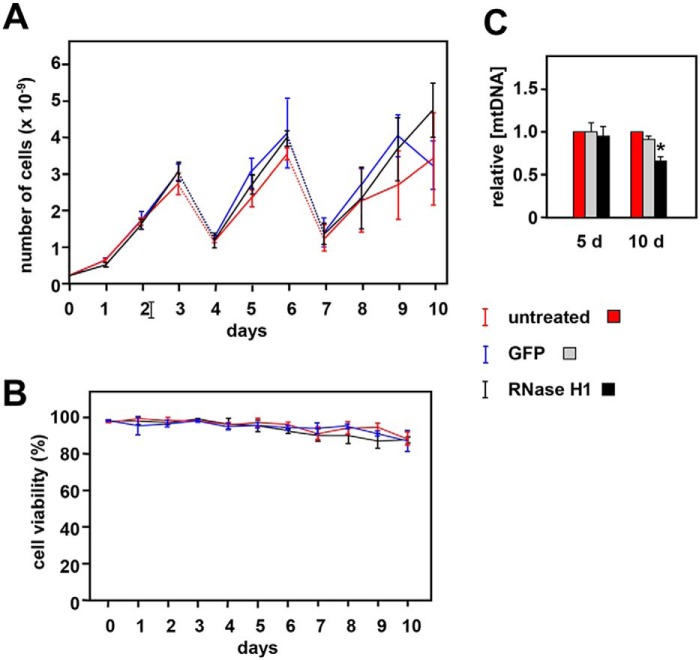
**Effects of *rnh1* knockdown in S2 cells.** *A*, growth curves for untreated S2 cells or cells treated with dsRNA against *rnh1* (RNase H1) or an inert dsRNA against GFP as indicated. Cells were split 1:6 on days (*d*) 3 and 6 with addition of fresh aliquots of dsRNA (*dotted lines*). Values are means for three independent replicates in each case. *Error bars* denote S.D. *B*, cell viability (trypan blue exclusion) for the same cell cultures. *C*, mean relative mtDNA copy number for the indicated cells, normalized against the value for untreated cells in parallel cultures. *Error bars* denote S.D. * denotes significant difference from untreated cells (Student's *t* test, *p* < 0.01, *n* = 3). Cells grown for 10 days were seeded at lower density (10^5^ cells/ml instead of the standard 10^6^/ml) with dsRNA added on days 0, 3, 5, and 8.

The prominent mitochondrial localization of epitope-tagged RNase H1 in S2 cells prompted us to analyze more closely the effects on mitochondria of manipulating its expression. We first verified the effectiveness and persistence of *rnh1* knockdown at the RNA level (Fig. S3*A*) and confirmed that there was no discernible effect on cell growth (Fig. 3*A*) or viability (Fig. 3*B*). Although 5 days of knockdown produced no significant mtDNA depletion (Ref. 43 and Fig. 3*C*), prolonged knockdown under optimal culture conditions did cause a significant drop in mtDNA copy number after 10 days (Fig. 3*C*).

**Figure 4.**
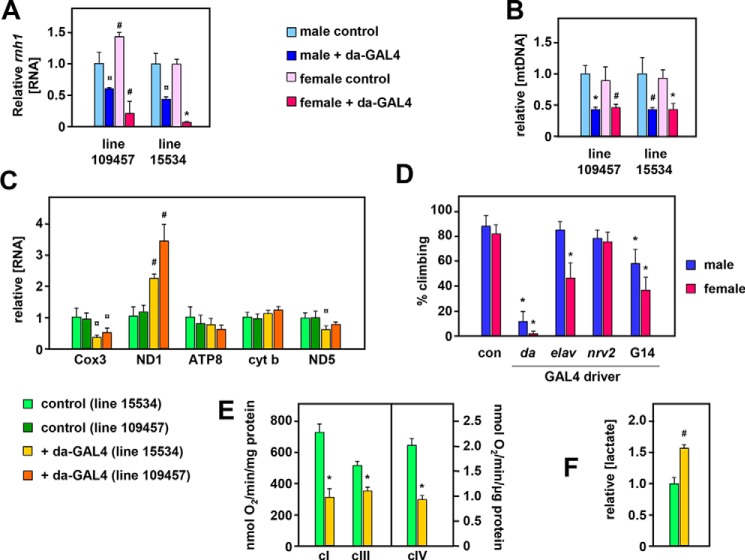
**Effects of *rnh1* knockdown in flies.** Relative *rnh1* transcript (*A*) and mtDNA copy number (mean ± S. D.) (*B*) in 2-day-old *rnh1* knockdown flies and corresponding controls (*i.e.* with balancer in place of the *da*-GAL4–bearing chromosome). Data are normalized in each case against male control flies. Significance values as indicated (Student's *t* test, comparing knockdown flies with controls of the given sex and RNAi strain, *n* = 4 in all cases). *C*, relative levels of transcripts (means + S.D.) of the indicated mitochondrial genes in 2-day-old female flies of the indicated knockdown strains *versus* balancer controls from the same strains. All data are normalized to values from controls of strain 15534. Significance values are as indicated (Student's *t* test, comparing knockdown flies of each RNAi strain with controls of the same strain, *n* = 4 in all cases). *D*, climbing index (54) of adult flies of the indicated sex, knocked down for *rnh1* (strain 15534) using the indicated GAL4 drivers. Values are means ± S.D. of nine independently tested batches of flies in each case. Significant differences are indicated (from control flies of the same sex; Student's *t* test). Note that because the *elav*-GAL4 driver is located on chromosome X, it was present only in female progeny; the males are therefore a control for this driver. *E*, oxygen consumption rates of mitochondrial suspensions from 10-day-old knockdown males of *da*-GAL4–driven RNAi line 15334 and controls of the same line, supplied with complex I (*cI*)-, III (*cIII*)-, and IV-linked (*cIV*) substrates. Values are means ± S.D. (*n* = 4, significant differences as shown between controls and knockdown flies). *F*, lactate levels in homogenates from 10-day-old knockdown males of *da*-GAL4–driven RNAi line 15334 and controls of the same line, normalized to protein content and then to the value from control flies. Values are means ± S.D. (*n* = 3, significant differences as shown, between controls and knockdown flies). All statistical comparisons are based on Student's *t* tests (unpaired, two-tailed) with *p* values denoted as ¤, *hash sign* (#), and * (<0.05, 0.01, and 0.001, respectively). *con*, control; *cyt b*, cytochrome *b*.

Because S2 cells are largely resistant to mitochondrial defects, including mtDNA depletion (43), and a null mutation in *rnh1* is lethal in flies, we proceeded to analyze the effects of *rnh1* knockdown at the whole-organism level using the ubiquitously acting *da*-GAL4 driver. Compared with controls lacking the driver, each of two knockdown lines eclosed in normal numbers (>90%; Fig. S4*A*) and with no significant change in development time (Fig. S4*B*). Knockdown at the RNA level was significant in both lines tested but appeared to be more severe in RNAi line 15534 than line 109457 and more pronounced in females than males (Fig. 4*A*). In both lines and sexes, we observed a >50% mtDNA depletion (Fig. 4*B*), which remained stable as the flies aged (Fig. S4*C*). Both lines also exhibited anomalies in mitochondrial transcript levels (Fig. 4*C*) with increased ND1 but decreased Cox3 RNA. To clarify the latter phenomenon, we confirmed the strandedness of transcription in each of the major gene clusters (Fig. S6).

Knockdown flies exhibited a severe locomotor defect (Movie S1), which was quantified by a negative geotaxis assay in flies of line 15534 (Fig. 4*D*) in which *rnh1* knockdown was driven either ubiquitously (*da*-GAL4 driver) or using drivers specific for neurons (*elav*-GAL4), glia (*nrv2*-GAL4), and muscle (G14-GAL4). The neuronal and muscle-specific drivers individually produced similar but milder locomotor defects than that produced by *da*-GAL4, suggesting that the latter is the result of additive or synergistic effects in the different tissues. Knockdown flies also showed hallmarks of mitochondrial dysfunction, such as respiratory chain impairment (Fig. 4*E*) and lactate accumulation (Fig. 4*F*), as well as shortened lifespan (Fig. S5).

### RNase H1 knockdown leads to the accumulation of abnormal mtDNA species

The effects of *rnh1* knockdown on mtDNA and RNA levels, combined with the organismal phenotype indicative of mitochondrial dysfunction, prompted us to look more closely at the molecular effects on mtDNA and its replication intermediates. qPCR does not distinguish between different topological forms of mtDNA or even between intact and damaged or partly fragmented molecules. Therefore, we used Southern blotting of purified mtDNA, treated with various modifying enzymes, to probe its structure.

**Figure 5.**
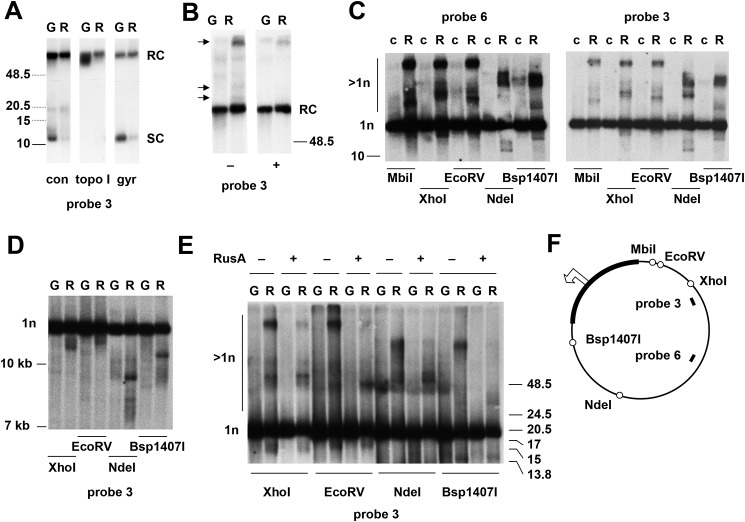
***rnh1* knockdown leads to the accumulation of abnormal mtDNA species.** *A–E*, Southern blots of mtDNA (mitochondrial nucleic acid) from control S2 cells (*c*) or S2 cells treated with an inert dsRNA against GFP (*G*) or a dsRNA against *rnh1* (*R*), probed as indicated. Migration of molecular weight markers (in kb) is indicated: *dotted* species in *A* are extrapolated from a separate gel of control material. *A*, undigested DNA, either untreated (*con*) or treated with topoisomerase I (*topo I*) or DNA gyrase (*gyr*). The forms inferred to be monomeric supercoiled circles (*SC*) or relaxed circles (*RC*) are as indicated. *B*, undigested DNA treated with (+) or without (−) S1 nuclease as shown. *Arrowheads* indicate molecular species unique to *rnh1* knockdown cells that were sensitive to S1 nuclease. The forms inferred to be monomeric relaxed circles (*RC*) are as indicated. *C* and *E*, DNA digested with restriction enzymes as shown. The forms inferred to be monomeric linears (1*n*) or species of higher apparent molecular weight (>1*n*) are as indicated. Samples in *E* were treated with (+) or without (−) RusA as indicated. *D*, DNA digested with restriction enzymes as shown. *F*, schematic map of *Drosophila* mtDNA, indicating the location of relevant restriction sites (*open circles*), the noncoding region (*bold*), and the probes used. The *open arrowhead* marks the location and direction of replication initiation (see Ref. 40), near which one of the double-stranded ends inferred in *D* maps.

The majority of the mtDNA from knockdown cells was intact, but several abnormalities were evident. First, the proportion of supercoiled molecules was decreased compared with control cells (Fig. 5*A*). Treatment with topoisomerase I converted the remaining molecules comigrating electrophoretically with supercoiled circles to the relaxed circular form, confirming that these molecules were indeed monomeric and supercoiled (Fig. 5*A*). Conversely, gyrase treatment, which converted a fraction of the relaxed circular monomers seen in control cells to the supercoiled form (Fig. 5*A*), had a much weaker effect on mtDNA from knockdown cells, indicating that most of it contained nicks (Fig. 5*A*). Mitochondrial DNA from knockdown cells also contained unique species migrating at high molecular weight that were differentially sensitive to S1 nuclease treatment (Fig. 5*B*, species indicated by *arrows*). Following digestion with a variety of restriction enzymes cutting once in the genome, we observed novel (or greatly enhanced) species of mtDNA from *rnh1* knockdown cells, some of which migrated more slowly (Fig. 5*C*), while others migrated faster (Fig. 5*D*) than linearized genomic monomers. Their mobility was consistent between sample preparations but varied according to the digest used. Some of the slow-migrating species were sensitive to the Holliday junction–specific resolvase RusA (Fig. 5*E*). This and their presence above the compression zone for linear molecules imply that they could represent branched molecules containing unresolved four-way junctions.

Based on their mobility (Fig. 5*D*), the faster-migrating species seen in *rnh1* knockdown cells indicate double-strand ends or fragile sites at several positions in the genome, one of which appears to lie close to the previously mapped replication origin (Refs. 40 and 41 and see Fig. 5*F*). This recalls previous data from mouse *Rnaseh1*-knockout cells (31) where fragile sites were attributed to nonremoval of RNA primers at the origin.

### RNase H1 knockdown produces mtDNA species consistent with fork regression

**Figure 6.**
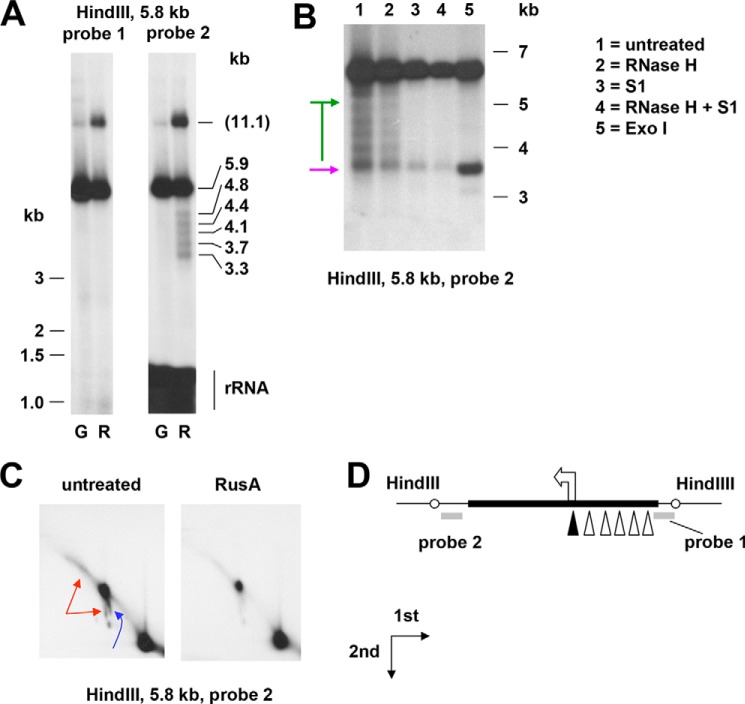
***rnh1* knockdown leads to fork regression.** *A–C*, Southern blots of mtDNA (mitochondrial nucleic acid) extracted from *rnh1* knockdown cells (*B*, *C*, and *lanes* denoted “*R*” in *A*) or control cells treated with an inert dsRNA against GFP (*lanes* denoted “*G*” in *A*), digested, and probed as indicated. In *A*, the migration of molecular weight markers of <3 kb is shown to the *left*, and extrapolated sizes of the bands are shown to the *right*, based on markers of >3 kb. Markers of 3 kb and above are shown to the *right* of *B*. Note that in the region below 1.5 kb, probe 2 also detected 16S rRNA. One-dimensional (*A* and *B*) and two-dimensional (*C*) agarose gels are shown. <1*n* fragments in *B* are denoted by a *purple arrow* (nuclease-resistant) or *green bar*/*arrows* (nuclease-sensitive). In *C*, higher molecular weight nonlinear forms are denoted by *red arrows* (flying, slow-moving Y-like arc) and *blue arrows* (burst bubbles and an arc in the shape of an apostrophe, extending downward from them). The directions of first- and second-dimension electrophoresis are as shown. *D*, schematic map of the region analyzed, showing location of probes (*bold gray lines*), NCR (*bold black line*), replication origin (*arrowhead*), and inferred ends of <1*n* fragments detected by probe 2 (*open triangles*; *filled triangle* for the nuclease-resistant fragment).

To investigate further the nature of the ends mapping close to the replication origin, we hybridized agarose gel blots of the NCR-containing HindIII fragment with probes located on either side of the NCR (Fig. 6). Probe 2, located in the rDNA region downstream of the origin, detected a set of shorter fragments (Fig. 6, *A* and *B*) with inferred termini located close to the origin and to sites upstream thereof, presumptively within each of the “four-and-a-half” copies of the repeat II element of the NCR (46, 47). None of these <1*n* fragments or any of reciprocal size (which would be in the size range of 1–2.5 kb) were detected by probe 1, lying on the other side of the NCR, indicating that they must be derived from nascent molecules rather than from a double-strand break or fragile site.

**Figure 7.**
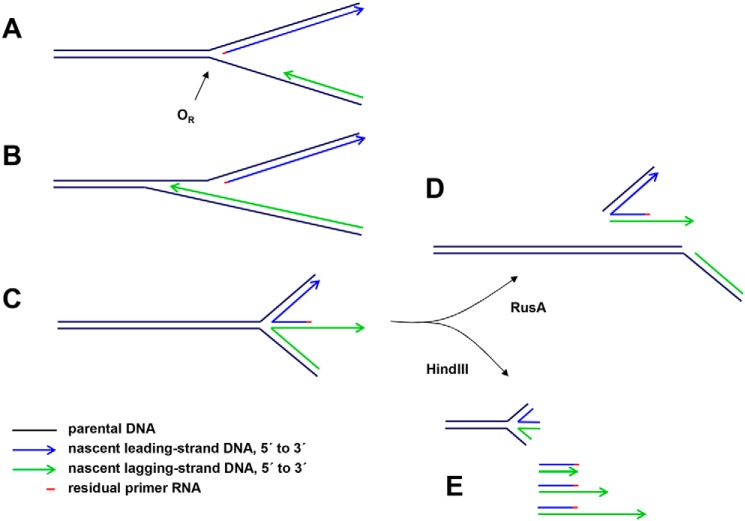
**Proposed interpretations of mtDNA species detected by gel electrophoresis in *rnh*1 knockdown cells.** One of many possible scenarios is illustrated. *A*, portion of a hypothetical replication intermediate with uncompleted lagging strand approaching the unidirectional replication origin (*O_R_*). A short residual RNA primer remains at the 5′ end of the leading strand. *B*, the lagging strand proceeds beyond the leading-strand initiation point as far as specific, reiterated termination signals in the repeat II elements of the NCR. *C*, impaired fork progression around the genome causes the origin structure to persist with eventual regression to form a chicken-foot structure that can branch-migrate (arc denoted by *blue arrow* in Fig. 6*C*). *D*, upon treatment with RusA, the four-way junctions resulting from these regressed forks are cut, generating effectively linear products. *E*, HindIII digestion liberates linear fragments with lagging-strand 3′ ssDNA extensions derived from the regressed forks (*green arrows* in Fig. 6*B*). These are digestible with S1 nuclease or exonuclease I, leaving a residual double-stranded species (*purple arrow* in Fig. 6*B*).

The longer <1*n* fragments were sensitive (Fig. 6*B*) to S1 nuclease and to exonuclease I (Exo I), indicating that they contain 3′ ssDNA extensions. The shortest of the bands was strongly enhanced by Exo I treatment (Fig. 6*B*), whereas the longest was also partially sensitive to RNase H (see Fig. 7 for interpretations).

A higher molecular weight fragment of apparent size 11.1 kb was observed to accumulate. 2DNAGE indicated that this material was nonlinear and digestible by RusA. Given its size and mobility, it should represent burst bubbles in which the replication fork has progressed beyond the end of the fragment but has not entered it from the other end to complete replication. Its sensitivity to RusA indicates also that it contains four-way junctions, which logically arise by fork regression from the origin, generating a chicken-foot structure that is able to branch migrate, generating the steeply descending arc observed in 2DNAGE (Fig. 6*C*, *blue arrow*). The 2DNAGE blot revealed, in addition, a “flying,” slow-moving Y-like arc (Fig. 6*C*, *red arrow*s), which may signal the presence of an undigested restriction site attributable to single strandedness or RNA/DNA hybrid, arising from failure to complete lagging-strand synthesis (20). Logically, the Exo I–sensitive extensions represent lagging strands terminating at specific sites within the repeat II region of the NCR. The structures detected in this analysis and how they may arise are depicted in Fig. 7.

### rnh1 knockdown leads to accumulation of specific replication intermediates

**Figure 8.**
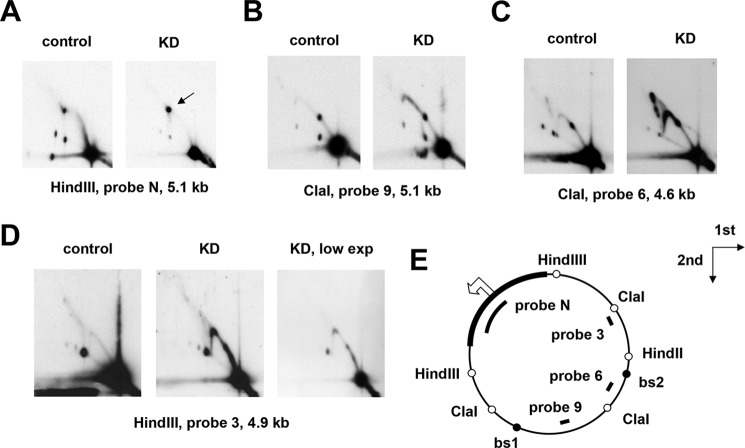
**Knockdown of *rnh1* cause accumulation of RIs beyond pause sites.** *A–D*, 2DNAGE of four restriction fragments of *Drosophila* S2 cell mtDNA, probed as indicated, in material from control cells treated with an inert dsRNA against GFP and cells knocked down for *rnh1* by treatment with an *rnh1*-specific dsRNA (denoted *KD*). The *arrow* in *A* denotes burst bubbles (see text). *E*, schematic map of *Drosophila* mtDNA indicating the location of relevant restriction sites (*open circles*), mTTF-binding sites (bs1 and bs2; *filled circles*), the noncoding region (*bold*), and the probes used. The *open arrowhead* marks the location and direction of replication initiation (see Ref. 40). The directions of the first- and second-dimension electrophoresis in all gels are as indicated by the *arrows*. The images show relatively low exposures (*low exp*) to reveal fine details of the arcs of RIs.

We infer from the preceding experiments that mtDNA replication was severely impaired in *rnh1* knockdown cells, sufficiently to result in eventual mtDNA depletion. To investigate more specifically the molecular defects caused by *rnh1* knockdown, we analyzed mtDNA replication intermediates (RIs) from around the mitochondrial genome using 2DNAGE. All fragments of the genome tested showed clear deviations in the pattern of RIs in knockdown cells compared with control cells (Fig. 8), indicating replication stalling or slowing in specific genomic regions. Accumulations of RIs were prominent in the regions immediately beyond the two major replication pause sites in the direction of fork progression (Fig. 8, *B* and *C*). These pauses are also the binding sites for the mitochondrial transcription termination factor (MTERF)-related protein mTTF (48). The observed regions of RI accumulation were quite discrete, extending ∼1.7 kb beyond the mTTF-binding site 1 (bs1) through the center of the ClaI fragment detected by probe 9 (Fig. 8*B*) and ∼5 kb beyond bs2 through the middle of the adjacent HindIII fragment. The latter zone exhibited internal regions of higher RI accumulation, revealed at low exposure (Fig. 8*D*). We confirmed that similar effects on RIs were seen in mtDNA from *rnh1* knockdown flies (Fig. S7).

Three other features may be noted. First, prominent junctional intermediates were detected in the ClaI fragment containing bs2 (X spike in Fig. 8*C*). Second, in the origin/terminus fragment, the bubble arc was weak or missing (Fig. 8*A*). Third, burst bubbles, in which replication forks have migrated beyond the end of the fragment but not yet entered from its other side, were enhanced relative to unreplicated mtDNA (Fig. 8*A*, species marked by an *arrow*).

### RNase H1 overexpression has effects on mtDNA replication opposite to those of RNase H1 knockdown

To evaluate the effects of overexpression of (epitope-tagged) RNase H1, we created stably transfected cell lines using the copper-inducible expression vector pMT-V5/HisB. Upon induction, RNase H1–overexpressing cells grew normally for 4–5 days but then entered growth arrest (Fig. S8*A*) with loss of cell viability (Fig. S8*B*). A control expressing a mitochondrially targeted fluorescent protein maintained viability and grew almost at the same rate as S2 cells that had not been induced with copper, expressing only the blasticidin resistance coselection marker (Fig. S8*A*). Cells expressing the uniquely nuclearly targeted M1V variant entered the growth and viability crisis slightly earlier than those expressing the natural protein (Fig. S8*C*), whereas cells expressing the predominantly mitochondrially targeted M16V variant entered the growth crisis approximately 2 days later (Fig. S8*C*). Nuclear overexpression is therefore sufficient to account for the growth and cell-viability defect conferred by RNase H1. However, to avoid any interference from this phenomenon, we proceeded to analyze mtDNA RIs from cells that had been grown for only 48 h following the induction of overexpression.

**Figure 9.**
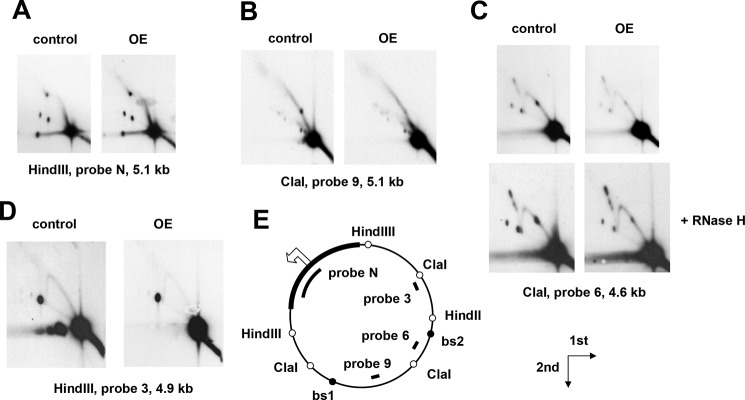
**Overexpression of *rnh1* relieves replication pausing.** *A–D*, 2DNAGE of four restriction fragments of *Drosophila* S2 cells mtDNA, probed as indicated, in material from control cells and cells overexpressing RNase H1 in the form of epitope-tagged RNase H1-V5 (denoted *OE*), both treated with 500 μm CuSO_4_ for 48 h to induce expression. *E*, schematic map of *Drosophila* mtDNA, as also shown in Fig. 8, indicating the location of relevant restriction sites (*open circles*), mTTF-binding sites (bs1 and bs2; *filled circles*), the noncoding region (*bold*), and the probes used. The *open arrowhead* marks the location and direction of replication initiation (see Ref. 40). The directions of first- and second-dimension electrophoresis in all gels are as indicated by the *arrows*. The images show relatively low exposures to reveal fine details of the arcs of RIs.

We observed clear alterations in the patterns of RIs detected by 2DNAGE (Fig. 9) upon overexpression of RNase H1. In most respects, the changes were opposite in nature to those produced by RNase H1 knockdown. RIs in most regions of the genome were diminished relative to the unreplicated fragment. Replication pauses were either abolished (Fig. 9*B*, bs1) or decreased (Fig. 9*C*, bs2), whereas the origin/terminus fragment and the adjacent HindIII fragment lacking pause sites were largely unaffected.

## Discussion

In this study, we determined that RNase H1 in *Drosophila*, as in mammals, is dually targeted to the nucleus and to mitochondria based on alternate AUG translation starts and the presence of mitochondrial and nuclear localization signals (Figs. 1 and 2). Targeting to both compartments was confirmed by microscopy and Western blotting for both transiently and stably transfected cells. Knockdown of *rnh1* in flies produced a phenotype characteristic of mitochondrial dysfunction (Figs. 4, S4, and S5) with respiratory chain deficiency, increased lactate, locomotor impairment, and curtailed lifespan. These accompanied mtDNA depletion and abnormal mitochondrial transcript levels. These findings led us to undertake a detailed study of the effects of manipulating RNase H1 expression on mtDNA replication intermediates. In S2 cells, *rnh1* knockdown (Fig. 8) and overexpression (Fig. 9) produced reciprocal changes in mtDNA replication intermediates with knockdown resulting in the accumulation of several abnormal mtDNA species (Figs. 5 and 6), the significance of which is discussed below.

### Global effects of RNase H1 manipulation on cell and organismal phenotype

Previous studies in *Drosophila* (44) and in mouse (30) implied that null mutations of the gene encoding RNase H1 are recessive-lethal. Dual targeting raises the question of whether this lethality is a nuclear or mitochondrial effect. In mouse embryos deleted for *Rnaseh1*, lethality coincided with drastic mtDNA depletion as for null mutations in other mtDNA maintenance genes, such as TFAM (5) or DNA polymerase γ (33). Using RNAi in *Drosophila* (Figs. 4, S4, and S5) we were able to show that RNase H1 also has a predominantly mitochondrial function in adults. This is probably the case in mammals as well: in the mouse, liver degeneration caused by hepatic *Rnaseh1* knockout was accompanied by mitochondrial dysfunction (49), and a point mutation in *RNASEH1* produces a recognizable mitochondrial disease phenotype in humans (28, 29). In contrast, *rnh1* knockdown in S2 cells had no obvious effect on cell growth or viability (Fig. 3, *A* and *B*). This accords with previous studies indicating that S2 cells do not depend on mitochondrial functions for survival (43) but indicates that nuclearly localized RNase H1 is also dispensable under the conditions of cell culture. In contrast, RNase H1 overexpression, specifically in the nucleus, was lethal after approximately 1 week of culture (Fig. S8). Overall, our results indicate that RNase H1 expression has to be tightly regulated in place and amount.

### Similarities between effects of nuclear and mitochondrial RNase H1 depletion

The effects on mitochondrial RNA and DNA synthesis of *rnh1* knockdown in S2 cells are similar to those previously inferred in both yeast (35, 50) and mammalian cells (36) in the nuclear compartment where it is required for the clearance of persistent heteroduplexes. We found an accumulation of mtDNA RIs in which the fork has progressed beyond the major replication pause sites (Fig. 8) into gene clusters that are heavily transcribed in the opposite direction (Fig. S6*B*), implying slow movement or stalling of replication forks in these regions. Furthermore, abnormalities in transcript levels imply that transcription and/or RNA processing is also disturbed. An interference with both replication fork movement and transcription is consistent with an accumulation of unresolved heteroduplexes in cells deficient for RNase H1. This indicates a role for RNase H1 in clearing such heteroduplexes, similar to its proposed function in the nucleus and in yeast mitochondria (51).

In addition, these findings could implicate RNase H1 as a component of the previously hypothesized machinery that handles conflicts between the replication and transcription machineries by regulating replication pausing at binding sites for MTERF and related proteins (13, 24). An involvement of RNase H1 with the pausing machinery is supported by the opposite effects of *rnh1* knockdown and overexpression on replication pausing (Figs. 8 and 9). RNase H1 overexpression caused a decrease in pausing, similar to the effect produced by overexpression of the major mitochondrial helicase (47), whereas RNase H1 knockdown caused increased pausing and fork stalling or slowing in the regions beyond the pause sites. Improperly regulated fork progression from pause sites may result in collisions with an oncoming transcription complex, leading to fork arrest and collapse.

Although its deficiency disturbed both mtDNA replication and transcription, it remains an open question whether RNase H1 functions as an integral component of the relevant machinery or as a stand-alone enzyme, processing heteroduplexes that would otherwise impair these processes. In other systems, RNase H1 has been found to interact with single strand–binding proteins (52, 53) and with gyrase (55). It will be interesting to see whether this also applies to the proteins of the mtDNA replisome in *Drosophila*.

### Fork regression and processing in RNase H1–deficient cells

Fork stalling at various points in the genome, whether as a result of unresolved heteroduplex or collisions, has various consequences. First, either a restart mechanism should be required to complete replication, or else the products of aborted replication need to be degraded and replaced by new synthesis. Second, repeated stalling and restart (or degradation) should decrease the rate of completion of each round of mtDNA replication. This may account not only for mtDNA depletion but also for the persistence of unprocessed initiation structures at the origin. In addition, the latter may be influenced directly by RNase H1 deficiency, as in mammals (31), if the enzyme is required for primer removal.

The abnormal mtDNA species detected in *rnh1* knockdown cells included those apparently joined by four-way (X) junctions (Fig. 5) based on their sensitivity to RusA (56). These may represent a signature of homology-driven restart but could also arise from fork regression or from a failure of segregation as in mammals (29). X-junctions were especially abundant in the region beyond mTTF-binding site 2 (Fig. 8*C*). Note that such junctions may have formed elsewhere in the genome but could have accumulated in specific regions following branch migration.

The other major abnormal species detected in *rnh1* knockdown cells contained a double-strand end at or near the replication origin. Some of these molecules also had 3′ ssDNA extensions of the lagging strand (Fig. 6). These forms could result from lagging-strand termination at specific, reiterated sites in the repeat II region of the NCR followed by the formation of chicken-foot structures that can then regress by branch migration (Fig. 7) as observed previously in the nucleus (57–59).

### Perspectives

Although there is still no consensus regarding the mechanisms of mtDNA replication in animals, the evidence from this and other studies (27–31) of a multiplicity of functions for RNase H1 is consistent with the general importance of RNA in mtDNA maintenance. As indicated above, the persistence of RNA/DNA hybrid is detrimental to DNA and RNA syntheses in many systems.

Elucidating the exact molecular function(s) of RNase H1 must await detailed study of its template preferences and interaction partners both *in vivo* and *in vitro. Drosophila* S2 cells, where the enzyme is needed to maintain mtDNA homeostasis but where mtDNA-encoded functions are not required for cell survival, should provide an excellent system in which to take this study forward.

## Experimental procedures

### Drosophila lines and cell culture

S2 cells (Invitrogen) were cultured as described (60) using Schneider's medium (Sigma). Standard WT (Oregon R), balancer, recipient (*w1118*), and driver (*da*-GAL4) *Drosophila* lines, originally sourced from stock centers, were maintained as described previously (61). RNAi lines 15534 (GD P-element library) and 109457 (KK φC31 library) for *rnh1* as well as the corresponding control line 60100 for the KK library were obtained from the Vienna *Drosophila* Resource Centre (VDRC). Note that RNAi line 15534 is no longer available from VDRC but is equivalent to line 15533, which is still maintained. The work required no ethical permit for the use of experimental animals because the use of invertebrates such as *Drosophila* is not covered by any relevant legislation in Finland.

### dsRNA treatment

Gene-specific dsRNA was produced with MEGAscript® T7 kit (Thermo Fisher Scientific) according to the manufacturer's instructions. Primers to create the templates for dsRNA production are as shown in Table S1. Knockdown using dsRNA against *rnh1* or an inert dsRNA against GFP as control was performed as follows. For 5-day knockdown experiments, cells were seeded at a density of 10^6^ cells/ml and treated with 8 μg/ml dsRNA for 30 min after plating. An additional 8 μg/ml dsRNA was added after 3 days. For 10-day knockdown, 10^5^ cells/ml were seeded and treated 0.5 h later with 8 μg/ml dsRNA and with a further 8 μg/ml dsRNA at days 3, 5, and 8 when medium was also refreshed.

### Plasmid constructs and transfection

S2 cell cDNA was synthesized as described previously (13) and used as template to amplify the *rnh1* mRNA sequence using chimeric primers to add restriction sites for EcoRI and XhoI, respectively, up- and downstream of the coding sequence (Table S1) but omitting the stop codon to create an in-frame fusion to the V5 epitope and oligohistidine tags of the cloning vector pMT-V5/HisB (Thermo Fisher Scientific). Note that the construct also included the short upstream ORF that may be important for regulated intracellular targeting based on data from studies of mammalian RNase H1 (27). Mutant versions were created by PCR using this construct as template together with oppositely oriented primers engineered to introduce the M1V or M16V mutation (Table S1). For M16V, the two primers were immediately adjacent in the sequence to retain the natural reading frame. For M1V, the reverse primer was situated upstream of the first methionine, and the forward primer overlapped 10 bp upstream and 10 bp downstream of the first methionine, which it converted from ATG to GTG. “Isoform B” and ΔNLS versions were each created in a two-step process. In the first step, segments up- and downstream of the region to be deleted were amplified separately but using primers with a 5-bp overlap. The products were then combined in an equimolar ratio for second-step amplification, deleting exon 2 or the putative nuclear localization signal (RKRK, amino acids 139–142), respectively, creating isoform B and ΔNLS variants via cloning into pMT-V5/HisB as above. All constructs were sequence-verified before use. Transfection of plasmid constructs into S2 cells was performed using FuGENE® (Promega). Transient expression of V5-tagged proteins was induced by adding CuSO_4_ to a final concentration of 500 μm. Two days after induction, cells were fixed and stained as described (62) using mouse anti-V5 (Life Technologies) and rabbit anti-COX IV (Abcam) as primary antibodies and Alexa Fluor® 568 goat anti-mouse IgG (H+L) and Alexa Fluor 488 goat anti-rabbit IgG (H+L) (Life Technologies) as secondary antibodies, mounted using ProLong Gold antifade medium with DAPI (Thermo Fisher Scientific), and imaged by confocal microscopy. To establish cell clones stably expressing V5-tagged RNase H1 and variants, pCoBlast (Thermo Fisher Scientific) was included in transfections. Two days after transfection, 20 ng/ml blasticidin (InvivoGen) was added to transfected cells in 3 ml of medium, and cells were maintained under selection for 3 weeks with harvesting every 3–4 days by centrifugation for 5 min at 1,000 × *g*_max_ at room temperature followed by resuspension in 3 ml of fresh, blasticidin-containing medium. Surviving cells were then cloned by plating at limiting dilution in 96-well plates in medium containing 20 ng/ml blasticidin. Expression of the tagged protein upon induction was verified by immunocytochemistry.

### Cell growth and viability

Aliquots of 10^5^ cells in 1 ml of medium were seeded into 12-well plates, and where appropriate, expression was induced by the addition of 500 μm CuSO_4_, or knockdown was obtained by the addition of 8 μg/ml gene-specific dsRNA. Three independent cultures of cells were each counted daily in triplicate using a hemocytometer, and the proportion of dead cells was determined by trypan blue exclusion. Every 3 days cells were diluted back to 10^5^ cells/ml after measurement with addition of fresh CuSO^4^ or dsRNA as appropriate.

### Cell synchronization and cell-cycle analysis

Cells stably transfected with RNase H1-V5 were seeded in 3 ml of medium at 5 × 10^5^ cells/ml, and expression was induced by the addition of 500 μm CuSO_4_. The next day, cells were synchronized in G_1_ or G_2_, respectively, with 0.5 mm hydroxyurea (Sigma) or 0.5 nm ponasterone A (Invitrogen) or left untreated. After 24 h, 1-ml aliquots of cells from each treatment were used for immunocytochemistry, and an additional 1-ml aliquot from each was pelleted at 1,000 × *g*_max_ for 5 min at 4 °C, washed in ice-cold PBS, then stained for 30 min on ice in the dark with 500 μl of propidium iodide staining solution containing 25 μg/ml propidium iodide, 100 μg/ml RNase A, 0.1% sodium citrate, and 0.1% Triton X-100 (Sigma). After 30 min, samples were analyzed by flow cytometry (488-nm excitation, >670-nm emission; FL3). The number of cells was plotted against the DNA content at each time point.

### Nucleic acid extraction and analysis

Total RNA was extracted from S2 cells, L3 larvae, pupae, and adult flies as described earlier (13), and cDNA was synthesized using a High-capacity cDNA Reverse Transcription kit (Thermo Fisher Scientific) according to the manufacturer's instructions. The cDNA was used as template in PCRs to analyze the representation of *rnh1* splice isoforms, using primers indicated in Table S1, with products detected by 1.5% agarose gel electrophoresis (1.7 V/cm) in Tris/Borate/EDTA buffer. For transcript quantitation, total RNA was isolated from S2 cells or adult flies, and cDNA was produced as described previously (13). Expression levels were determined by qRT-PCR in an Applied Biosystems StepOnePlus^TM^ Real-Time PCR System with a Fast SYBR^TM^ Green Master Mix kit from Applied Biosystems, using as template 2 μl of cDNA product diluted 10-fold in a 20-μl reaction together with 10 pmol of each gene-specific primer pair for *rnh1* or mtDNA protein-coding genes ATP8, cytochrome *b*, Cox3, ND1, and ND5 (see Table S1; reactions carried out as for Surf1 mRNA in Ref. 62). Transcript levels were normalized against RpL32. For strand-specific qRT-PCR, the same process was followed as above, but during cDNA synthesis, random primers were substituted by gene-specific sense or antisense primer for each mitochondrial gene analyzed, cytochrome *b*, Cox2, ND5, and 16S rRNA (primers as detailed in Table S1), as well as for RpL32 (antisense strand). mtDNA copy number analysis from S2 cell total DNA isolation and qPCR were performed as described previously (43). For mtDNA copy number analysis from adult *Drosophila*, 500 μl of prelysis buffer (75 mm NaCl, 50 mm EDTA, and 20 mm HEPES/NaOH, pH 7.8) was added to a single fly in an Eppendorf tube followed by crushing using a plastic pestle. Following the addition of 5 μl of 20% SDS and 400 μg of Proteinase K (Thermo Fisher Scientific), samples were incubated for 4 h at 50 °C with regular resuspension of the debris using the pestle. After allowing the debris to settle, the cleared homogenate was pipetted into a fresh Eppendorf tube, and crude nucleic acid was precipitated overnight at −20 °C following the addition of 420 μl of isopropanol. After centrifugation at 16,000 × *g*_max_ for 30 min at 4 °C, the DNA pellet was dissolved overnight in TE buffer (10 mm Tris/HCl and 1 mm EDTA, pH 7.8) at 55 °C. mtDNA was quantified by qPCR using primers against ND5 with RpL32 as an internal standard (see Table S1) in a StepOnePlus Real-Time PCR System with a Fast SYBR Green Master Mix kit.

To analyze mtDNA topology, integrity, and replication intermediates, mitochondrial nucleic acid was prepared from S2 cells, adult flies, and L3 larvae as described previously (13). For topology analysis, 1-μg aliquots of mitochondrial nucleic acid were incubated with the following enzymes, in 30 μl of manufacturer-supplied reaction buffer at 37 °C except where stated, and conditions as follows: topoisomerase I (New England Biolabs), 2 units, 30 min; gyrase (Topogen), 2 units, 60 min; restriction endonucleases MbiI, XhoI, EcoRV, NdeI, and Bsp1407I (Thermo Fisher Scientific), 4 units, 4 h; RNase H (Thermo Fisher Scientific), 0.5 unit, 60 min; S1 nuclease (Thermo Fisher Scientific), 2 units, 2 min at room temperature; RusA as described previously (13); and exonuclease I (Thermo Fisher Scientific), 10 units, 60 min. Reactions were deproteinized by phenol-chloroform extraction, and 2 μl of the aqueous phase was analyzed by 0.5% agarose gel electrophoresis (30-cm gels run for 40 h at 1.7 V/cm in Tris/Borate/EDTA). Where successive digestions with enzymes requiring different conditions were required, reactions were scaled up to 2 μg of DNA in 100 μl. After phenol-chloroform extraction, DNA was recovered from 95 μl of the aqueous phase by the addition of 9.5 μl of 3 m ammonium acetate and 240 μl of 99% ethanol, overnight precipitation at −20 °C, and centrifugation at 16,000 × *g*_max_ for 20 min at 4 °C. Pellets were air-dried for 20 min at room temperature and then resuspended in 20 μl of water. After measuring the concentration of the recovered nucleic acid, the second digestion was performed as described above.

For analysis of replication intermediates, restriction endonuclease digestion and 2DNAGE were carried out as described previously (13). Southern hybridization to *Drosophila* mtDNA probes 3, 6, or 9, or to probes N, 1, and 2 generated similarly (see Table S1), was performed as described previously (13). Filter membranes were exposed to Hyperfilm ECL X-ray film (Amersham Biosciences) for between 1 and 10 days.

### Developmental time and eclosion analysis

*da*-GAL4/TM3 Sb females were crossed with the RNAi line or VDRC control males in vials (12 females and four males). After 24 h of egg laying, adult flies were removed, and vials were incubated at 25 °C. The number of flies eclosing on each day was counted and used to calculate a mean eclosion day for each vial. To compute the eclosion rate, the total number of eclosed flies was divided by the number of pupae. This procedure was repeated four times for each cross.

### Climbing (negative geotaxis) assay

RNAi line 15534 females were crossed to driver or *w1118* males in bottles. Ten eclosing flies of each sex from each cross were placed in (separate) food vials and aged for 5 days. Each such batch of flies was transferred to an empty vial with a line drawn at a height of 6 cm. Vials were gently tapped to collect flies at the bottom, and the number of flies climbing above the 6 cm line within 10 s was recorded. This was repeated six times (technical replicates to generate an average score per vial) with the entire procedure repeated three times to generate biological replicates.

### Lifespan

*da*-Gal4/TM3 Sb females were crossed with RNAi line males or males from the corresponding control line (*w1118* or the VDRC KK control line) in bottles. Of the progeny, 20 males and 20 virgin females were randomly collected and transferred to vials in five replicates. Dead flies were removed daily, and their number was recorded. Flies were transferred to fresh food vials three times per week. Lifespan curves were generated by plotting the number of surviving flies on each day.

### Respirometry

Batches of 100 adult male flies of a given genotype were pooled into one food bottle and allowed to recover from CO_2_ exposure for at least for 24 h. Animals were transferred into a chilled mortar and homogenized carefully on ice in 500 μl of homogenization buffer (250 mm sucrose, 5 mm Tris/HCl, and 2 mm EGTA, pH 7.4) with 30 gentle pestle strokes. Lysates were filtered through 200-μm nylon mesh and rinsed with 500 μl of homogenization buffer at 4 °C. Oxygen consumption of 25-μl aliquots of the fly lysates was measured using an oxygen electrode apparatus (Hansatech Oxytherm) containing 475 μl of respiration buffer (120 mm KCl, 5 mm KH_2_PO_4_, 3 mm HEPES/KOH, 1 mm EGTA, 1 mm MgCl_2_, and 0.2% BSA, pH 7.2) with sequential additions of substrates and inhibitors to the following final concentrations: proline (10 mm), pyruvate (10 mm), ADP (1 mm), rotenone (150 nm), glycerol 3-phosphate (10 mm), antimycin (0.1 μm), ascorbate (10 mm) plus *N*,*N*,*N*,*N*-tetramethyl-*p*-phenylenediamine (10 mm), and KCN (200 μm). To minimize the effects of fluctuations in the performance of the apparatus on different days, equal numbers of control and knockdown fly lysates were analyzed on each day.

### Lactate measurements

Batches of 20 adult male flies were homogenized in 100 μl of PBS on ice in an Eppendorf tube and incubated at 65 °C for 15 min. Debris was pelleted by a short centrifugation (acceleration to 10,000 × *g*_max_ followed by immediate deceleration), and 10 μl of each lysate was assayed using BioVision kit K607 according to the manufacturer's protocol. A standard curve was generated using lactate concentrations of 100–1000 pmol.

### Subcellular protein fractionation and Western blotting

Subcellular fractionation to obtain nuclear and mitochondrial fractions without significant cross-contamination was implemented as follows. Cells were seeded at 10^6^ cells/ml in 100 ml of medium in a 175-cm^2^ flask. Expression of epitope-tagged RNase H1-V5 was induced with 500 μm CuSO_4_ over 2 days. Cells were harvested by centrifugation at 1,000 × *g*_max_ for 5 min at 4 °C, washed with 10 ml of ice-cold PBS, and finally resuspended in 2 ml of ice-cold STM buffer (250 mm sucrose, 50 mm Tris/HCl, 5 mm MgCl_2_, and 1× cOmplete^TM^ protease inhibitor (Roche Applied Science), pH 7.4). After 15 min of incubation on ice, cells were Dounce-homogenized with 20 strokes of a tight-fitting pestle, after which lysis was checked by trypan blue exclusion, with additional strokes to complete the process if required. Crude nuclei were pelleted by centrifugation at 800 × *g*_max_ for 15 min at 4 °C. Both pellet and supernatant were saved. To further purify nuclei, the pellet was resuspended in 2 ml of ice-cold STM buffer and recentrifuged at 800 × *g*_max_ for 15 min at 4 °C, and the supernatant was discarded. This step was repeated five times, after which the final nuclear pellet was lysed by resuspension in 1 ml of ice-cold NET buffer (20 mm HEPES/KOH, 1.5 mm MgCl_2_, 0.5 m NaCl, 0.2 mm EDTA, 20% glycerol, 1% Triton X-100, and 1× cOmplete protease inhibitor, pH 7.9) and treated with 10 units of DNase I (Thermo Fisher Scientific) for 30 min at 37 °C. Insoluble debris was removed by centrifugation at 1,000 × *g*_max_ for 10 min at 4 °C, after which nuclear components were pelleted by centrifugation at 14,000 × *g*_max_ for 10 min at 4 °C. The pellet was resuspended in 100 μl of PBS, heated to 95 °C for 10 min, and saved for analysis. For purification of the mitochondrial fraction, the original saved supernatant from pelleting of crude nuclei was recentrifuged twice at 1,000 × *g*_max_ for 10 min at 4 °C with the final supernatant recentrifuged at 11,000 × *g*_max_ for 10 min at 4 °C. The resulting pellet was washed with 1 ml of ice-cold STM buffer, resuspended in 200 μl of ice-cold STM, and layered over a 4-ml 1 m, 1.5 m sucrose step gradient in 20 mm HEPES/KOH, pH 7.2. The gradient was centrifuged at 180,000 × *g*_max_ for 60 min at 4 °C in a swinging bucket rotor (Beckman SW60Ti), and mitochondria were carefully recovered from the interface, diluted to 2 ml with HB buffer (225 mm mannitol, 75 mm sucrose, 10 mm Tris/HCl, 1 mm EDTA, and 0.1% BSA, pH 7.6), and recovered by centrifugation at 14,000 × *g*_max_ for 10 min at 4 °C. The pellet was then lysed in 100 μl of lysis buffer (50 mm Tris/HCl, 150 mm NaCl, 1 mm EDTA, and 1% Triton X-100, pH 7.4) containing 1× cOmplete protease inhibitor. After incubation on ice for 30 min, the sample was centrifuged at 14,000 × *g*_max_ for 5 min at 4 °C, and the supernatant was saved for later analysis. Aliquots (20 μg) of the protein extracts were electrophoresed on SDS, 12% polyacrylamide gels; wet-blotted to nitrocellulose membranes (GE Healthcare); and processed for Western blotting using standard methods with reaction buffer containing 0.1% Tween 20 plus 5% BSA except for the final washes. Primary antibodies were against COX IV (Abcam, ab16056; rabbit polyclonal, 1:5,000), histone H4 (Abcam, ab10158; rabbit polyclonal, 1:10,000), and V5 (Invitrogen, R96025; mouse monoclonal, 1:10,000). Secondary antibodies were IRDye 680LT donkey anti-rabbit IgG (LI-COR Biosciences) and IRDye 680LT donkey anti-mouse IgG (LI-COR Biosciences), both used at 1:10,000. Blots were visualized using an LI-COR Odyssey imaging system and analyzed with Image Studio Live, version 4.0.

### Image processing

Gel images were optimized for brightness and contrast, cropped, and in some cases resized for clarity and comparability, but no other manipulations were carried out.

## Author contributions

J. M. G. d. C., M. G., E. T., E. D., H. T. J., and P. J. conceptualization; J. M. G. d. C., M. G., E. T., J. G., E. D., H. T. J., and P. J. formal analysis; J. M. G. d. C., M. G., E. T., E. D., H. T. J., and P. J. supervision; J. M. G. d. C., H. T. J., and P. J. funding acquisition; J. M. G. d. C. and H. T. J. validation; J. M. G. d. C., M. G., E. T., J. G., E. D., and P. J. investigation; J. M. G. d. C., M. G., E. T., J. G., E. D., H. T. J., and P. J. methodology; J. M. G. d. C. and H. T. J. writing-original draft; J. M. G. d. C., H. T. J., and P. J. project administration; J. M. G. d. C., M. G., E. T., J. G., E. D., H. T. J., and P. J. writing-review and editing.

## Supplementary Material

Supporting Information

## Footnotes

3 The abbreviations used are:

mtDNA: mitochondrial DNA
Pol: polymerase
TFAM: mitochondrial transcription factor A
2DNAGE: two-dimensional neutral agarose gel electrophoresis
NCR: noncoding region
NLS: nuclear localization signal
qPCR: quantitative PCR
Exo: exonuclease
ssDNA: single-stranded DNA
RI: replication intermediate
mTTF: mitochondrial transcription termination factor (MTERF)-related protein
bs: binding site
VDRC: Vienna *Drosophila* Resource Centre
Cox: cytochrome *c* oxidase
DAPI: 4′,6-diamidino-2-phenylindole
qRT-PCR: quantitative RT-PCR.

## References

[1] Ciesielski G. L., Oliveira M. T., and Kaguni L. S. (2016) Animal mitochondrial DNA replication. Enzymes 39, 255–292 10.1016/bs.enz.2016.03.006 27241933 PMC4964852

[2] Kasiviswanathan R., Collins T. R., and Copeland W. C. (2012) The interface of transcription and DNA replication in the mitochondria. Biochim. Biophys. Acta 1819, 970–978 10.1016/j.bbagrm.2011.12.005 22207204 PMC3334424

[3] Young M. J., and Copeland W. C. (2016) Human mitochondrial DNA replication machinery and disease. Curr. Opin. Genet. Dev. 38, 52–62 10.1016/j.gde.2016.03.005 27065468 PMC5055853

[4] Shutt T. E., and Gray M. W. (2006) Bacteriophage origins of mitochondrial replication and transcription proteins. Trends Genet. 22, 90–95 10.1016/j.tig.2005.11.007 16364493

[5] Larsson N. G., Wang J., Wilhelmsson H., Oldfors A., Rustin P., Lewandoski M., Barsh G. S., and Clayton D. A. (1998) Mitochondrial transcription factor A is necessary for mtDNA maintenance and embryogenesis in mice. Nat. Genet. 18, 231–236 10.1038/ng0398-231 9500544

[6] Stiles A. R., Simon M. T., Stover A., Eftekharian S., Khanlou N., Wang H. L., Magaki S., Lee H., Partynski K., Dorrani N., Chang R., Martinez-Agosto J. A., and Abdenur J. E. (2016) Mutations in TFAM, encoding mitochondrial transcription factor A, cause neonatal liver failure associated with mtDNA depletion. Mol. Genet. Metab. 119, 91–99 10.1016/j.ymgme.2016.07.001 27448789

[7] Fisher R. P., and Clayton D. A. (1988) Purification and characterization of human mitochondrial transcription factor 1. Mol. Cell. Biol. 8, 3496–3509 10.1128/MCB.8.8.3496 3211148 PMC363587

[8] Wang Y. E., Marinov G. K., Wold B. J., and Chan D. C. (2013) Genome-wide analysis reveals coating of the mitochondrial genome by TFAM. PLoS One 8, e74513 10.1371/journal.pone.0074513 23991223 PMC3753274

[9] Kang D., Kim S. H., and Hamasaki N. (2007) Mitochondrial transcription factor A (TFAM): roles in maintenance of mtDNA and cellular functions. Mitochondrion 7, 39–44 10.1016/j.mito.2006.11.017 17280879

[10] Alam T. I., Kanki T., Muta T., Ukaji K., Abe Y., Nakayama H., Takio K., Hamasaki N., and Kang D. (2003) Human mitochondrial DNA is packaged with TFAM. Nucleic Acids Res. 31, 1640–1645 10.1093/nar/gkg251 12626705 PMC152855

[11] Matsushima Y., Garesse R., and Kaguni L. S. (2004) *Drosophila* mitochondrial transcription factor B2 regulates mitochondrial DNA copy number and transcription in Schneider cells. J. Biol. Chem. 279, 26900–26905 10.1074/jbc.M401643200 15060065

[12] Watanabe A., Arai M., Koitabashi N., Niwano K., Ohyama Y., Yamada Y., Kato N., and Kurabayashi M. (2011) Mitochondrial transcription factors TFAM and TFB2M regulate Serca2 gene transcription. Cardiovasc. Res. 90, 57–67 10.1093/cvr/cvq374 21113058

[13] Jõers P., Lewis S. C., Fukuoh A., Parhiala M., Ellilä S., Holt I. J., and Jacobs H. T. (2013) Mitochondrial transcription terminator family members mTTF and mTerf5 have opposing roles in coordination of mtDNA synthesis. PLoS Genet. 9, e1003800 10.1371/journal.pgen.1003800 24068965 PMC3778013

[14] Fan L., Kim S., Farr C. L., Schaefer K. T., Randolph K. M., Tainer J. A., and Kaguni L. S. (2006) A novel processive mechanism for DNA synthesis revealed by structure, modeling and mutagenesis of the accessory subunit of human mitochondrial DNA polymerase. J. Mol. Biol. 358, 1229–1243 10.1016/j.jmb.2006.02.073 16574152 PMC4703138

[15] Farge G., Pham X. H., Holmlund T., Khorostov I., and Falkenberg M. (2007) The accessory subunit B of DNA polymerase γ is required for mitochondrial replisome function. Nucleic Acids Res. 35, 902–911 10.1093/nar/gkl1116 17251196 PMC1807957

[16] Humble M. M., Young M. J., Foley J. F., Pandiri A. R., Travlos G. S., and Copeland W. C. (2013) Polg2 is essential for mammalian embryogenesis and is required for mtDNA maintenance. Hum. Mol. Genet. 22, 1017–1025 10.1093/hmg/dds506 23197651 PMC3561914

[17] Maier D., Farr C. L., Poeck B., Alahari A., Vogel M., Fischer S., Kaguni L. S., and Schneuwly S. (2001) Mitochondrial single-stranded DNA-binding protein is required for mitochondrial DNA replication and development in *Drosophila melanogaster*. Mol. Biol. Cell 12, 821–830 10.1091/mbc.12.4.821 11294889 PMC32269

[18] Sugimoto T., Mori C., Takanami T., Sasagawa Y., Saito R., Ichiishi E., and Higashitani A. (2008) *Caenorhabditis elegans* par2.1/mtssb-1 is essential for mitochondrial DNA replication and its defect causes comprehensive transcriptional alterations including a hypoxia response. Exp. Cell Res. 314, 103–114 10.1016/j.yexcr.2007.08.015 17900564

[19] Yang M. Y., Bowmaker M., Reyes A., Vergani L., Angeli P., Gringeri E., Jacobs H. T., and Holt I. J. (2002) Biased incorporation of ribonucleotides on the mitochondrial L-strand accounts for apparent strand-asymmetric DNA replication. Cell 111, 495–505 10.1016/S0092-8674(02)01075-9 12437923

[20] Yasukawa T., Reyes A., Cluett T. J., Yang M. Y., Bowmaker M., Jacobs H. T., and Holt I. J. (2006) Replication of vertebrate mitochondrial DNA entails transient ribonucleotide incorporation throughout the lagging strand. EMBO J. 25, 5358–5371 10.1038/sj.emboj.7601392 17066082 PMC1636616

[21] Pohjoismäki J. L., Holmes J. B., Wood S. R., Yang M. Y., Yasukawa T., Reyes A., Bailey L. J., Cluett T. J., Goffart S., Willcox S., Rigby R. E., Jackson A. P., Spelbrink J. N., Griffith J. D., Crouch R. J., et al (2010) Mammalian mitochondrial DNA replication intermediates are essentially duplex but contain extensive tracts of RNA/DNA hybrid. J. Mol. Biol. 397, 1144–1155 10.1016/j.jmb.2010.02.029 20184890 PMC2857715

[22] Miralles Fusté J., Shi Y., Wanrooij S., Zhu X., Jemt E., Persson Ö., Sabouri N., Gustafsson C. M., and Falkenberg M. (2014) *In vivo* occupancy of mitochondrial single-stranded DNA binding protein supports the strand displacement mode of DNA replication. PLoS Genet. 10, e1004832 10.1371/journal.pgen.1004832 25474639 PMC4256270

[23] Fusté J. M., Wanrooij S., Jemt E., Granycome C. E., Cluett T. J., Shi Y., Atanassova N., Holt I. J., Gustafsson C. M., and Falkenberg M. (2010) Mitochondrial RNA polymerase is needed for activation of the origin of light-strand DNA replication. Mol. Cell 37, 67–78 10.1016/j.molcel.2009.12.021 20129056

[24] Shi Y., Posse V., Zhu X., Hyvärinen A. K., Jacobs H. T., Falkenberg M., and Gustafsson C. M. (2016) Mitochondrial transcription termination factor 1 directs polar replication fork pausing. Nucleic Acids Res. 44, 5732–5742 10.1093/nar/gkw302 27112570 PMC4937320

[25] Reyes A., Kazak L., Wood S. R., Yasukawa T., Jacobs H. T., and Holt I. J. (2013) Mitochondrial DNA replication proceeds via a 'bootlace' mechanism involving the incorporation of processed transcripts. Nucleic Acids Res. 41, 5837–5850 10.1093/nar/gkt196 23595151 PMC3675460

[26] Holt I. J., Kazak L., Reyes A., and Wood S. R. (2016) Analysis of replicating mitochondrial DNA by *in organello* labeling and two-dimensional agarose gel electrophoresis. Methods Mol. Biol. 1351, 95–113 10.1007/978-1-4939-3040-1_8 26530677

[27] Suzuki Y., Holmes J. B., Cerritelli S. M., Sakhuja K., Minczuk M., Holt I. J., and Crouch R. J. (2010) An upstream open reading frame and the context of the two AUG codons affect the abundance of mitochondrial and nuclear RNase H1. Mol. Cell. Biol. 30, 5123–5134 10.1128/MCB.00619-10 20823270 PMC2953059

[28] Reyes A., Melchionda L., Nasca A., Carrara F., Lamantea E., Zanolini A., Lamperti C., Fang M., Zhang J., Ronchi D., Bonato S., Fagiolari G., Moggio M., Ghezzi D., and Zeviani M. (2015) RNASEH1 mutations impair mtDNA replication and cause adult-onset mitochondrial encephalomyopathy. Am. J. Hum. Genet. 97, 186–193 10.1016/j.ajhg.2015.05.013 26094573 PMC4572567

[29] Akman G., Desai R., Bailey L. J., Yasukawa T., Dalla Rosa I., Durigon R., Holmes J. B., Moss C. F., Mennuni M., Houlden H., Crouch R. J., Hanna M. G., Pitceathly R. D., Spinazzola A., and Holt I. J. (2016) Pathological ribonuclease H1 causes R-loop depletion and aberrant DNA segregation in mitochondria. Proc. Natl. Acad. Sci. U.S.A. 113, E4276–E4285 10.1073/pnas.1600537113 27402764 PMC4968715

[30] Cerritelli S. M., Frolova E. G., Feng C., Grinberg A., Love P. E., and Crouch R. J. (2003) Failure to produce mitochondrial DNA results in embryonic lethality in Rnaseh1 null mice. Mol. Cell 11, 807–815 10.1016/S1097-2765(03)00088-1 12667461

[31] Holmes J. B., Akman G., Wood S. R., Sakhuja K., Cerritelli S. M., Moss C., Bowmaker M. R., Jacobs H. T., Crouch R. J., and Holt I. J. (2015) Primer retention owing to the absence of RNase H1 is catastrophic for mitochondrial DNA replication. Proc. Natl. Acad. Sci. U.S.A. 112, 9334–9339 10.1073/pnas.1503653112 26162680 PMC4522766

[32] Hogrefe H. H., Hogrefe R. I., Walder R. Y., and Walder J. A. (1990) Kinetic analysis of *Escherichia coli* RNase H using DNA-RNA-DNA/DNA substrates. J. Biol. Chem. 265, 5561–5566 1690712

[33] Hance N., Ekstrand M. I., and Trifunovic A. (2005) Mitochondrial DNA polymerase gamma is essential for mammalian embryogenesis. Hum. Mol. Genet. 14, 1775–1783 10.1093/hmg/ddi184 15888483

[34] Wu H., Sun H., Liang X., Lima W. F., and Crooke S. T. (2013) Human RNase H1 is associated with protein P32 and is involved in mitochondrial pre-rRNA processing. PLoS One 8, e71006 10.1371/journal.pone.0071006 23990920 PMC3750045

[35] Shen W., Sun H., De Hoyos C. L., Bailey J. K., Liang X. H., and Crooke S. T. (2017) Dynamic nucleoplasmic and nucleolar localization of mammalian RNase H1 in response to RNAP I transcriptional R-loops. Nucleic Acids Res. 45, 10672–10692 10.1093/nar/gkx710 28977560 PMC5737507

[36] Parajuli S., Teasley D. C., Murali B., Jackson J., Vindigni A., and Stewart S. A. (2017) Human ribonuclease H1 resolves R-loops and thereby enables progression of the DNA replication fork. J. Biol. Chem. 292, 15216–15224 10.1074/jbc.M117.787473 28717002 PMC5602383

[37] Arora R., Lee Y., Wischnewski H., Brun C. M., Schwarz T., and Azzalin C. M. (2014) RNaseH1 regulates TERRA-telomeric DNA hybrids and telomere maintenance in ALT tumour cells. Nat. Commun. 5, 5220 10.1038/ncomms6220 25330849 PMC4218956

[38] Tannous E., Kanaya E., and Kanaya S. (2015) Role of RNase H1 in DNA repair: removal of single ribonucleotide misincorporated into DNA in collaboration with RNase H2. Sci. Rep. 5, 9969 10.1038/srep09969 25951507 PMC4423430

[39] Maul R. W., Chon H., Sakhuja K., Cerritelli S. M., Gugliotti L. A., Gearhart P. J., and Crouch R. J. (2017) R-loop depletion by over-expressed RNase H1 in mouse B cells increases activation-induced deaminase access to the transcribed strand without altering frequency of isotype switching. J. Mol. Biol. 429, 3255–3263 10.1016/j.jmb.2016.12.020 28065739 PMC5500445

[40] Jõers P., and Jacobs H. T. (2013) Analysis of replication intermediates indicates that *Drosophila melanogaster* mitochondrial DNA replicates by a strand-coupled theta mechanism. PLoS One 8, e53249 10.1371/journal.pone.0053249 23308172 PMC3537619

[41] Saito S., Tamura K., and Aotsuka T. (2005) Replication origin of mitochondrial DNA in insects. Genetics 171, 1695–1705 10.1534/genetics.105.046243 16118189 PMC1456096

[42] Clayton D. A. (1982) Replication of animal mitochondrial DNA. Cell 28, 693–705 10.1016/0092-8674(82)90049-6 6178513

[43] Fukuoh A., Cannino G., Gerards M., Buckley S., Kazancioglu S., Scialo F., Lihavainen E., Ribeiro A., Dufour E., and Jacobs H. T. (2014) Screen for mitochondrial DNA copy number maintenance genes reveals essential role for ATP synthase. Mol. Syst. Biol. 10, 734 10.15252/msb.20145117 24952591 PMC4265055

[44] Filippov V., Filippov M., and Gill S. S. (2001) *Drosophila* RNase H1 is essential for development but not for proliferation. Mol. Genet. Genomics 265, 771–777 10.1007/s004380100483 11523794

[45] Young S. K., and Wek R. C. (2016) Upstream open reading frames differentially regulate gene-specific translation in the integrated stress response. J. Biol. Chem. 291, 16927–16935 10.1074/jbc.R116.733899 27358398 PMC5016099

[46] Lewis D. L., Farr C. L., Farquhar A. L., and Kaguni L. S. (1994) Sequence, organization, and evolution of the A+T region of *Drosophila melanogaster* mitochondrial DNA. Mol. Biol. Evol. 11, 523–538 8015445 10.1093/oxfordjournals.molbev.a040132

[47] Ciesielski G. L., Nadalutti C. A., Oliveira M. T., Jacobs H. T., Griffith J. D., and Kaguni L. S. (2018) Structural rearrangements in the mitochondrial genome of *Drosophila melanogaster* induced by elevated levels of the replicative DNA helicase. Nucleic Acids Res. 46, 3034–3046 10.1093/nar/gky094 29432582 PMC5887560

[48] Roberti M., Polosa P. L., Bruni F., Musicco C., Gadaleta M. N., and Cantatore P. (2003) DmTTF, a novel mitochondrial transcription termination factor that recognises two sequences of *Drosophila melanogaster* mitochondrial DNA. Nucleic Acids Res. 31, 1597–1604 10.1093/nar/gkg272 12626700 PMC152874

[49] Lima W. F., Murray H. M., Damle S. S., Hart C. E., Hung G., De Hoyos C. L., Liang X. H., and Crooke S. T. (2016) Viable RNaseH1 knockout mice show RNaseH1 is essential for R loop processing, mitochondrial and liver function. Nucleic Acids Res. 44, 5299–5312 10.1093/nar/gkw350 27131367 PMC4914116

[50] Amon J. D., and Koshland D. (2016) RNase H enables efficient repair of R-loop induced DNA damage. Elife 5, e20533 10.7554/eLife.20533 27938663 PMC5215079

[51] El Hage A., Webb S., Kerr A., and Tollervey D. (2014) Genome-wide distribution of RNA-DNA hybrids identifies RNase H targets in tRNA genes, retrotransposons and mitochondria. PLoS Genet. 10, e1004716 10.1371/journal.pgen.1004716 25357144 PMC4214602

[52] Nguyen H. D., Yadav T., Giri S., Saez B., Graubert T. A., and Zou L. (2017) Functions of replication protein A as a sensor of R loops and a regulator of RNaseH1. Mol. Cell 65, 832–847.e4 10.1016/j.molcel.2017.01.029 28257700 PMC5507214

[53] Petzold C., Marceau A. H., Miller K. H., Marqusee S., and Keck J. L. (2015) Interaction with single-stranded DNA-binding protein stimulates *Escherichia coli* ribonuclease HI enzymatic activity. J. Biol. Chem. 290, 14626–14636 10.1074/jbc.M115.655134 25903123 PMC4505529

[54] Kemppainen K. K., Rinne J., Sriram A., Lakanmaa M., Zeb A., Tuomela T., Popplestone A., Singh S., Sanz A., Rustin P., and Jacobs H. T. (2014) Expression of alternative oxidase in *Drosophila* ameliorates diverse phenotypes due to cytochrome oxidase deficiency. Hum. Mol. Genet. 23, 2078–2093 10.1093/hmg/ddt601 24293544 PMC3959817

[55] Yang Z., Hou Q., Cheng L., Xu W., Hong Y., Li S., and Sun Q. (2017) RNase H1 cooperates with DNA gyrases to restrict R-loops and maintain genome integrity in *Arabidopsis* chloroplasts. Plant Cell 29, 2478–2497 10.1105/tpc.17.00305 28939594 PMC5774575

[56] Bolt E. L., and Lloyd R. G. (2002) Substrate specificity of RusA resolvase reveals the DNA structures targeted by RuvAB and RecG *in vivo*. Mol. Cell. 10, 187–198 10.1016/S1097-2765(02)00560-9 12150918

[57] Nelson S. W., and Benkovic S. J. (2010) Response of the bacteriophage T4 replisome to noncoding lesions and regression of a stalled replication fork. J. Mol. Biol. 401, 743–756 10.1016/j.jmb.2010.06.027 20600127 PMC2943651

[58] Bugreev D. V., Rossi M. J., and Mazin A. V. (2011) Cooperation of RAD51 and RAD54 in regression of a model replication fork. Nucleic Acids Res. 39, 2153–2164 10.1093/nar/gkq1139 21097884 PMC3064783

[59] Maric C., and Bénard M. (2014) Replication forks reverse at high frequency upon replication stress in *Physarum polycephalum*. Chromosoma 123, 577–585 10.1007/s00412-014-0471-z 24951952

[60] Schneider I. (1972) Cell lines derived from late embryonic stages of *Drosophila melanogaster*. J. Embryol. Exp. Morphol. 27, 353–365 4625067

[61] Fernandez-Ayala D. J., Sanz A., Vartiainen S., Kemppainen K. K., Babusiak M., Mustalahti E., Costa R., Tuomela T., Zeviani M., Chung J., O'Dell K. M., Rustin P., and Jacobs H. T. (2009) Expression of the *Ciona intestinalis* alternative oxidase (AOX) in *Drosophila* complements defects in mitochondrial oxidative phosphorylation. Cell Metab. 9, 449–460 10.1016/j.cmet.2009.03.004 19416715

[62] Rogers S. L., and Rogers G. C. (2008) Culture of *Drosophila* S2 cells and their use for RNAi-mediated loss-of-function studies and immunofluorescence microscopy. Nat. Protoc. 3, 606–611 10.1038/nprot.2008.18 18388942

