# Supplementary material for: RNase H1 promotes replication fork progression through oppositely transcribed regions of Drosophila mitochondrial DNA
Source: J Biol Chem. 2019 Jan 11;294(12):4331–44. doi: 10.1074/jbc.RA118.007015 (PMC6433063; doi:10.1074/jbc.RA118.007015)
Supplement: Supporting Information [file supp_294_12_4331__index.html]

RNase H1 promotes replication fork progression through oppositely transcribed regions of Drosophila mitochondrial DNA — RNase H1 and Drosophila mtDNA replication — RNase H1 promotes replication fork progression through oppositely transcribed regions of Drosophila mitochondrial DNA — RNase H1 and Drosophila mtDNA replication — Supporting Information 

# RNase H1 promotes replication fork progression through oppositely transcribed regions of *Drosophila* mitochondrial DNA

## Supporting Information

- Supporting Information (to be published online) - supplementary movie S1
- Supporting Information (to be published online) - Supplementary figures and their legends
