# Supplementary material for: RNase H1 promotes replication fork progression through oppositely transcribed regions of Drosophila mitochondrial DNA
Source: J Biol Chem. 2019 Jan 11;294(12):4331–44. doi: 10.1074/jbc.RA118.007015 (PMC6433063; doi:10.1074/jbc.RA118.007015)
Supplement: Supporting Information [file supp_RA118.007015_142391_1_supp_265299_pl2dlv.pdf]

RNase H1 promotes replication fork progression through oppositely transcribed regions of *Drosophila* mitochondrial DNA

Jose M. González de Cózar, Mike Gerards, Eveliina Teeri,

Jack George, Eric Dufour, Howard T. Jacobs & Priit Jõers

SUPPLEMENTARY DATA

SUPPLEMENTARY TABLES

Supplementary Table S1

Oligonucleotides used in the study

Name	Sequence (5´-3´)	Purpose
mtDNA_hybri_probe3F	AACTATTTTACCAGCAATTATTTTACT	mtDNA hybridization probe 3
mtDNA_hybri_probe3R	CAGTCATCTAATGAAGAGTTATTTCTA	mtDNA hybridization probe 3
mtDNA_hybri_probe6F	AAATCAATCAATTTAATATTCTACCTC	mtDNA hybridization probe 6
mtDNA_hybri_probe6R	ATTAACAATATTTATAGCTGGATTAGG	mtDNA hybridization probe 6
mtDNA_hybri_probe9F	AATCCATAAGATAATATATCACAACCT	mtDNA hybridization probe 9
mtDNA_hybri_probe9R	ATAATCTTATTTTTGATTTACAAGACC	mtDNA hybridization probe 9
NCRprobe_Fw	TAAATTTATTCCCCCTATTC	mtDNA hybridization probe N
NCRprobe_Rev	CATGATTTTATTATATAAATATTTTTTATAAAAATAATAC	mtDNA hybridization probe N
mtDNA_probe1F	AATGAATTGCCTGATAAAAAG	mtDNA hybridization probe 1
mtDNA_probe1R	TGTAGATATTAAATTATTATTA	mtDNA hybridization probe 1

mtDNA_probe2F	TAGAGCTAAAATCAAATTATT	mtDNA hybridization probe 2
mtDNA_probe2R	ATACCATTAATACAAATAAAT	mtDNA hybridization probe 2
RNAseH1 amp. RE fwd	TGATCATGGAATTCTGCCACATATGTTTCGATAGAC	cloning RNase H1 cDNA
RNAseH1 amp. RE rev	GTGAACGCTCGAACCATTTTTCTGCTTATACAAGG	cloning RNase H1 cDNA
RNaseH1met1.Fw	TTAGAAAATTGTGTTACTTCCGCG	Mutation M1V
RNaseH1met1.Rev	CGTTGTCTATCGAAACATATG	Mutation M1V
RNaseH1met16.Fw	CGCTGCACGGTGGCGTTTTAC	Mutation M16V
RNaseH1met16.Rev	TTGGAGGTTCCAGCAAAAATA	Mutation M16V
T7_dsRNA-GFP_R	GAATTAATACGACTCACTATAGGGAGAACGTAAACGGCCACA AGTTCAGC	Template for dsRNA production (GFP)
T7_dsRNA-GFP_F	GAATTAATACGACTCACTATAGGGAGAGGGTGTTCTGCTGGTA GTGGTCG	Template for dsRNA production (GFP)
T7_dsRNA-CG8729_F	GAATTAATACGACTCACTATAGGGAGAGCCAAATACAAGAAG	Template for dsRNA production (*rnh1*)
T7_dsRNA-CG8729_R	GAATTAATACGACTCACTATAGGGAGACAAACTGGAAGGCAC	Template for dsRNA production (*rnh1*)

NLSdel_extended_F	TCTCAATGGCACAACCAGCGGCGATAAGCGGAAC	NLS deleted RNase H1
NLSdel_extended_R	TTGTGCCATTGAGAATGTCTGGTAAATTGCTACT	NLS deleted RNase H1
ND5_forward	GGGTGAGATGGTTTAGGACTT	mtDNA copy number analysis
ND5_reverse	AAGCTACATCCCCAATTCGAT	mtDNA copy number analysis
RpL32_f	TGTGCACCAGGAACTTCTTGAA	mtDNA copy number analysis (nuclear DNA normalization standard), qRT-PCR and strand-specific qRT-PCR
RpL32_r	AGGCCCAAGATCGTGAAGAA	mtDNA copy number analysis (nuclear DNA normalization standard), qRT-PCR and strand-specific qRT-PCR
RNAseH1 2exon Fw	GTGGCCCGAAGAGGATCACGACCT	Analyze presence of second exon in *Drosophila rnh1* A/B transcript
RNAseH1 2exon Rev	ACCTGAGAGGCATGACGTGGGATC	Analyze presence of second exon in *Drosophila rnh1* A/B transcript
MT-ATP8 fwd	ATTCCACAAATAGCACCTATTAGATG	qRT-PCR for ATP8
MT-ATP8 rev	TTCATTAGATTTAGGTGAATTTGGT	qRT-PCR for ATP8
MT-CYTB fwd	ACCTGCCCATATTCAACCAG	qRT-PCR for cyt b

MT-CYTB rev	TGAATCCCTCGGAATTTTCTT	qRT-PCR for cyt b
MT-COXIII fwd	CGAGAAGGAACATACCAAGGA	qRT-PCR for COXIII
MT-COXIII rev	GCGGGTGATAAACTTCTGTGA	qRT-PCR for COXIII
ND1_f	GAAGAGGGGGTTTGGCTTTA	qRT-PCR for ND1
ND1_r	AACGAAATCGAGGTAAAGTTCC	qRT-PCR for ND1
MT-ND5 fwd	CAAGAAGAAAAAGGAATCTGAGC	qRT-PCR for ND5
MT-ND5 rev	TCGAATTGGGGATGTAGCTT	qRT-PCR for ND5
CG8729_f	AGGTCTCAGAAGCCACAGGA	qRT-PCR for *rnh1*
CG8729_f	CTGGTGATTCTTGCCGAAAT	qRT-PCR for *rnh1*
16S For	ACCTGGCTTACACCGGTTTG	cDNA synthesis and strand-specific qRT-PCR for 16S rRNA sense strand
16S Rev	GGGTGTAGCCGTTCAAATTT	cDNA synthesis and strand-specific qRT-PCR for 16S rRNA antisense strand
Cox2 for	AAAGTTGACGGTACACCTGGA	cDNA synthesis and strand-specific qRT-PCR for Cox2 antisense strand
Cox2 rev	TGATTAGCTCCACAGATTTC	cDNA synthesis and strand-specific qRT-PCR for Cox2 sense strand

ND5 for	GGGTGAGATGGTTTAGGACTTG	cDNA synthesis and strand-specific qRT-PCR for ND5 antisense strand
ND5 rev	AAGCTACATCCCCAATTCGAT	cDNA synthesis and strand-specific qRT-PCR for ND5 sense strand
CytB For	GAAAATTCCGAGGGATTCAA	cDNA synthesis and strand-specific qRT-PCR for cyt b antisense strand
CytB Rev	AACTGGTCGAGCTCCAATTC	cDNA synthesis and strand-specific qRT-PCR for cyt b sense strand

LEGENDS TO SUPPLEMENTARY FIGURES

Figure S1

Analysis of splice isoforms of *rnh1* transcripts

(A) Scale map of predicted transcripts from the RNase H1-encoding *rnh1* gene of *D. melanogaster,* from flybase.org. Top line depicts genomic DNA (gDNA) of the region. Coding regions shown in orange, non-coding regions in grey, introns as black boxes or as thin black lines joining exonic RNA segments, 3´ terminal exons terminating as arrowheads in direction of transcription. The adjacent gene, *drosha*, is transcribed in the opposite direction, as indicated. Black arrowheads denote the locations of PCR primers used to detect both of the predicted splice isoforms rnh1-RA and rnh1-RB. (B) Agarose gel analysis of PCR products from three biological replicate cDNAs from S2 cells, third larval instar (L3), pupa and adult males and female flies, alongside control products corresponding to the predicted splice isoforms as indicated, which were derived from cDNA expression plasmids created for the purpose. M - molecular weight markers. Predicted isoform rnh1-RB was undetectable in all samples, even though by virtue of its PCR product being shorter, it should have been preferentially amplified. Note also the very faint 'ghost-bands' of higher molecular weight. Although they might represent minor splice isoforms, no such forms have been reported in transcriptome databases, including those derived from high-content RNAseq analyses. Thus, it is much more likely that they are due to minor cross-reaction of the primers elsewhere in the transcriptome, and can be discounted. This experiment revealed isoform rnh1-RA to be the sole detectable *rnh1* mRNA in both cells and flies, and this was therefore studied in all subsequent experiments.

Figure S2

Supplementary information on subcellular localization of RNase H1

(A) Alignment of translational start regions of RNase H1 cDNAs from *D. melanogaster* (Dros, NCBI NM_057967.5, commencing at nt 1) and *M. musculus* (Mouse, NCBI NM_011275.3, commencing at nt 59), alongside implied amino acid sequences of the encoded polypeptides. Dashes indicate gaps introduced to maximize alignment. In both species, the first AUG specifies only a short upstream ORF, shown in purple, which is unrelated to RNase H1 protein, but is considered a signature of translational regulation (45). RNase H1 itself is encoded by a longer ORF, commencing with the AUG designated as methionine codon M1, shown in red, with a second translation start (methionine codon M16 in the *Drosophila* protein), shown, together with the rest of the coding sequence, in blue. RNase H1-V5 variant cDNAs M1V and M16V were created by modifying the respective start codons (underlined) to valine-encoding GTG. (B) Probabilities of targeting to mitochondria of the two hypothetical *D. melanogaster* RNase H1 polypeptides, 'Red' commencing at the first major translation start, and 'Blue' commencing at the second, as in (A), based on three commonly used prediction programmes as indicated. (C) Full polypeptide sequence of *D. melanogaster* RNase H1, showing in blue the hypothetical polypeptide commencing at the second in-frame translation start, in red the upstream region unique to the polypeptide commencing at the first translation start and, highlighted in yellow, the run of four basic residues (at the core of the strongest bipartite nuclear localization signal predicted by cNLS Mapper (http://nls-mapper.iab.keio.ac.jp/cgi-bin/NLS_Mapper_form.cgi), underlined. To create the ΔNLS variant, these four basic residues were deleted. The predicted cleavage site for the mitochondrial presequence

(RRS/GV) is boxed. (D) Representative images from immunocytochemistry of cells transiently transfected with RNase H1-V5 N-terminal methionine variants M1V and M16V, probed for the V5 epitope tag (red), cytochrome oxidase subunit 4 (Cox4, green), and DAPI (blue). M16V was localized mainly in mitochondria, but with some cells, e.g. that shown at top left, exhibiting faint nuclear localization. M1V was exclusively nuclear. For summary of results for many cells see Fig. 2A. (E) Cropped images of Western blots of total cell extracts (R), probed for epitope-tag V5, from cells transiently transfected with constructs expressing RNase H1-V5 variants as indicated. M - molecular weight markers sizes as indicated. Doublet bands, where present, are visible in the images when magnified. (F) FACS analysis for DNA content of unsynchronized control cells, cells arrested in G1 by hydroxyurea treatment, and arrested in G2 by ponasterone A treatment. Cells were stably transfected for RNase H1-V5 and had been induced to express the protein for 24 h prior to and then throughout the experiment, by culture in 500 μM $CuSO_4$. The major peaks corresponding with cells in cell-cycle phases G1 and G2 (plus mitosis, M), based on DNA content, with the region in between the peaks representing cells in S phase, as indicated above the first panel.

Figure S3

RNAi knockdown of *rnh1* transcripts in S2 cells

qRT-PCR analysis of relative level of *rnh1* transcript in cells treated with an inert dsRNA against GFP, a dsRNA against *rnh1*, or untreated control cells, as shown, over 9 days, all normalized to the value in untreated cells at day 0. Means ± SD of three replicates in each case.

Figure S4

Supplementary information on effects of *rnh1* knockdown in flies

(A) Proportion of pupae eclosing from crosses in which the dsRNA transgene targeted against *rnh1* was expressed ubiquitously under the control of the *da*-GAL4 driver, using two different RNAi lines and controls as indicated. (B) Mean eclosion day $\pm$SD of flies of the indicated sex and genotype. Controls have balancer in place of the *da*-GAL4-bearing chromosome. All progeny flies were heterozygous for the RNAi construct. (C) Relative mtDNA copy number during adult life of *rnh1* knockdown flies and corresponding controls (i.e. with balancer in place of the *da*-GAL4-bearing chromosome). Data are normalized in each case against the mtDNA level in control flies of the given sex and strain, one day after eclosion. Note that knockdown flies from line 15534 were shorter-lived than those from line 109457 (see Fig. S5), so time points at which copy number was assessed differ between the lines. Significance values as indicated (Student's *t* test, n = 5 in all cases, for comparisons berween knockdown and control flies of a given strain and age).

Figure S5

Lifespan curves of *rnh1* knockdown flies and controls

Lifespan curves of flies of the indicated sex and genotype, of RNAi line 15534, plus the w^{1118} recipient line as control.

Figure S6

Supplementary information on strandedness of transcription in *Drosophila* mtDNA

(A) Summary map of *Drosophila* mtDNA, arbitrarily linearized for clarity, showing

non-coding region (bold), origin and direction of replication (open arrowhead, Ref. 40), locations of mTTF binding sites bs1 and bs2 (filled circles), and gene clusters according to coding strand ('red' coding strand oppositely oriented to the direction of replication, 'blue' coding strand oriented as the direction of replication), named according to the representative gene whose expression was analysed by strand-specific qRT-PCR The positions and coding strand of these genes are as indicated. Second line shows *Cla*I and *Hin*dIII restriction sites relevant to the study. (B) Strand-specific qRT-PCR for the 4 indicated genes. Fold differences were calculated from raw ΔCt values (mean $\pm$ SD, n=3) for the 'blue' and 'red' strand transcripts, and plotted, as shown, on a logarithmic scale. ΔCt values for the two strands were significantly different from each other in every case ($p < 0.001$, Student's *t* test).

Figure S7

Effects of *rnh1* knockdown on mtDNA replication intermediates in L3 larvae

2DNAGE of *Cla*I-digested mtDNA from wild-type and *rnh1* knockdown larvae, hybridized with probe 6. Directions of first and second dimension electrophoresis as shown. Compare with the equivalent gels from S2 cells (Fig. 8C).

Figure S8

Effects of RNase H1-V5 (over)expression in S2 cells

(A) Growth curves for S2 cells stably transfected with pCoBlast vector, or with expression plasmids for mitochondrially targeted YPF (mtYFP) or RNase H1-V5, induced to express by treatment with 500 μM $CuSO_4$. Cells were split 1:6 on days 3 and 6, with addition of fresh $CuSO_4$ (dotted lines). Means $\pm$ SD for 3 independent replicates in each case. (B) Cell viability (trypan blue exclusion) for wild-type S2

cells, and cells stably transfected with expression plasmids for mitochondrially targeted YPF (mtYFP) or RNase H1-V5, induced to express by treatment with 500 μM $CuSO_4$. Cells were split 1:6 on days 3 and 6, with addition of fresh $CuSO_4$. Means ± SD for 3 independent replicates in each case. (C) Viability of cells similarly expressing RNase H1-V5 variants M1V and M16V. Note that the curves shown in panels (B) and (C) were from the same experiment, and have been separated here only for clarity. Cells expressing the M1V variant reached 50% viability after approximately 5.5 days of culture, whilst those expressing the wild-type variant did so about half a day later, and those expressing M16V only after approximately 8.5 days. M1V cells also reached <20% viability approximately 2 days earlier than cells expressing the wild-type variant. Although a toxic effect from mitochondrial over-expression cannot be ruled out, this experiment demonstrates that nuclear over-expression of RNase H1 is sufficient to compromize cell growth and viability.

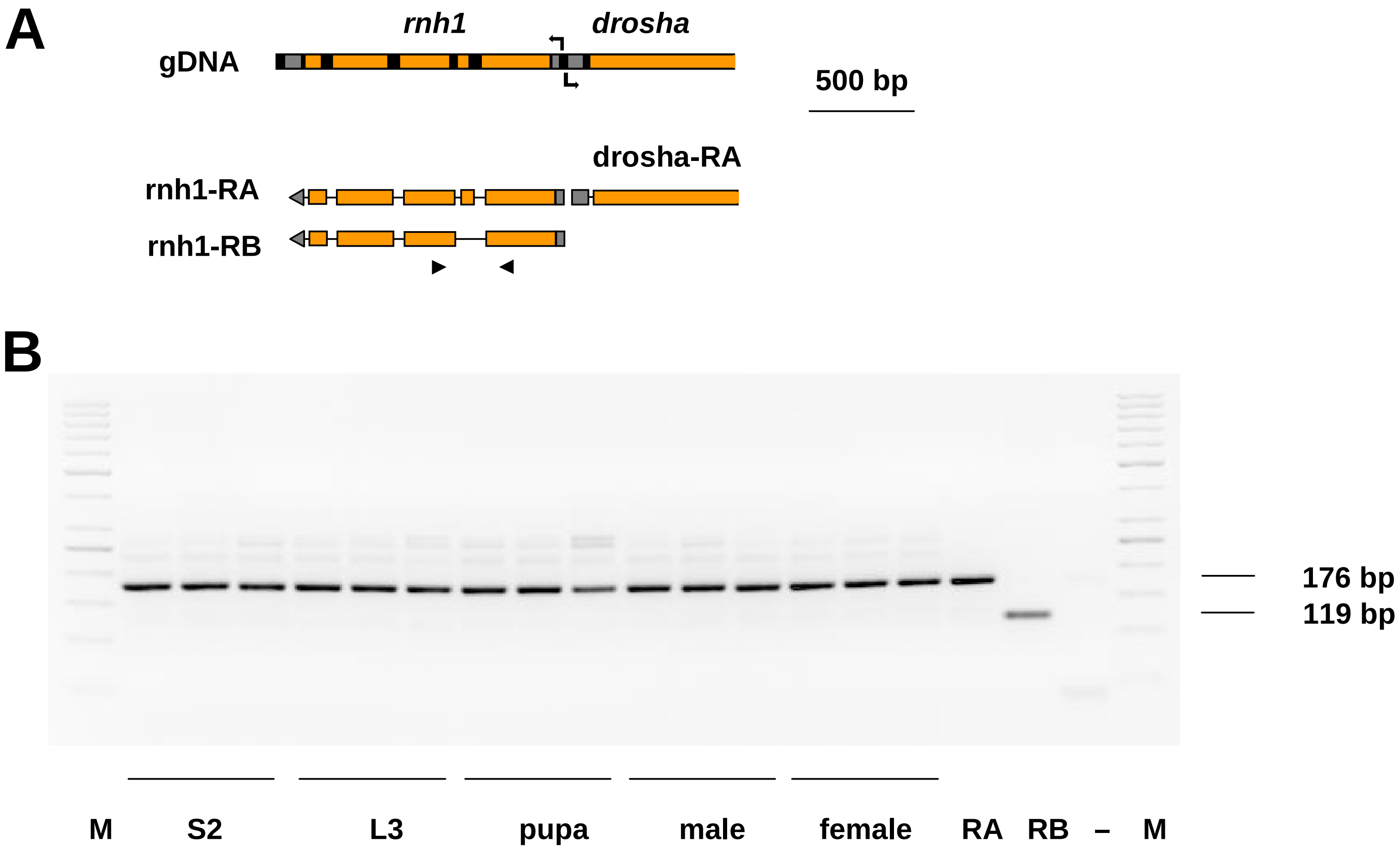

González de Cózar et al, Figure S1

A

```
            M  F  R  *                 M  L  L  P  R  Y  F  C  W  N  L  Q  R  C  T
Dros     CATATGTTTCGATAGACAACGTAAGAAAATTATGTTACTTCCGCGGTATTTTTGCTGGAACCTCCAACGCTGCACG
Mouse    ..CAATGTGGGTTGCCGCGCTGGCGTGAGTGATGCGCTGGCTGCTGCCGCTGTCCCGCACAGTGACACTGGCCGTT
            M  W  V  A  A  L  A  *     M  R  W  L  L  P  L  S  R  T  V  T  L  A  V
```

```
         -  -  -  -  -  -  -  -  -  -  -  M  A  F  Y  A  V  A  S  G  R  R  S  G
Dros     ---------------------------------ATGGCGTTTTACGCTGTAGCCAGTGGGCGGCGGTCTGGA...
Mouse    GTACGCCTGAGGCGAGGCATTTGCGGGCTCGGGATG---TTCTATGCGGTGAGGAGAGGCCGCAGGACCGGA...
         V  R  L  R  R  G  I  C  G  L  G  M  -  F  Y  A  V  R  R  G  R  R  T  G
```

B

	Red	Blue
TargetP	71%	30%
PSORT	39%	8.7%
MitoProt	93%	70%

C

```
MLLPRYFCWNLQRCTMAFYAVASGRRSGVYGSWAECEEQVKGFKNAKYKKFKTRQEADQFVNGCKSYAPQDVAVPLGKEK
ASLASWKNSIEVNKNPKYTDEWPEEDHDLAEDDLNAAMNEVEGDPKPSNSSNLPDILNRKRKGTTSGDKRNKIPRHASQV
SEATGLKQVGAFQFEIDAEGYVIVYTDGSCIGNGRAGACAGYGVYFGKNHQLNAAKPVEGRVTNNVGEIQAAIHAIKTAL
DLGIQKLCISTDSQFLINSITLWVAGWKKRDWKLKNNQPVKNVVDFKELDKLLQENNITVKWNYVEAHKGIEGNEMADKL
ARQGSALYKQKNG
```

D

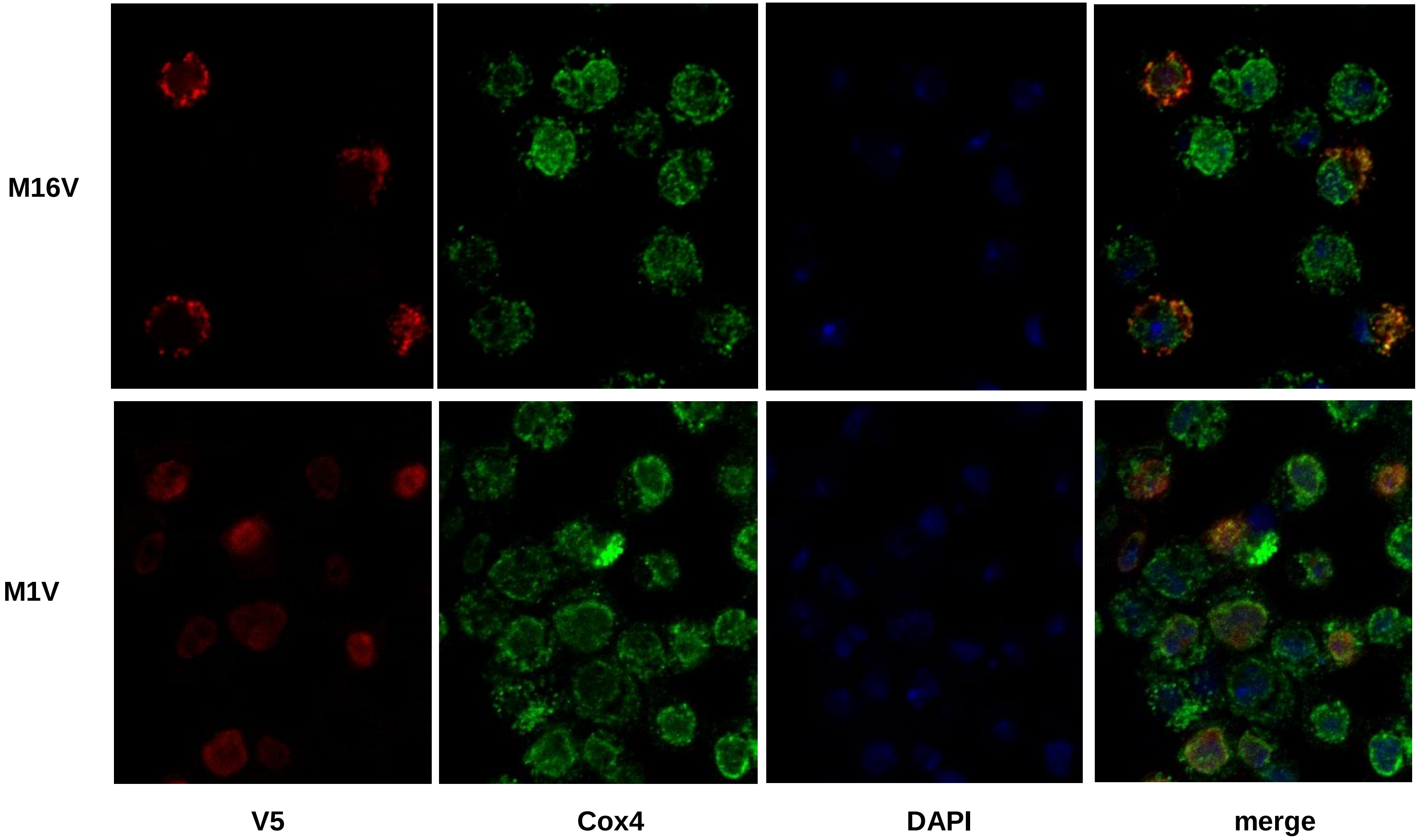

E

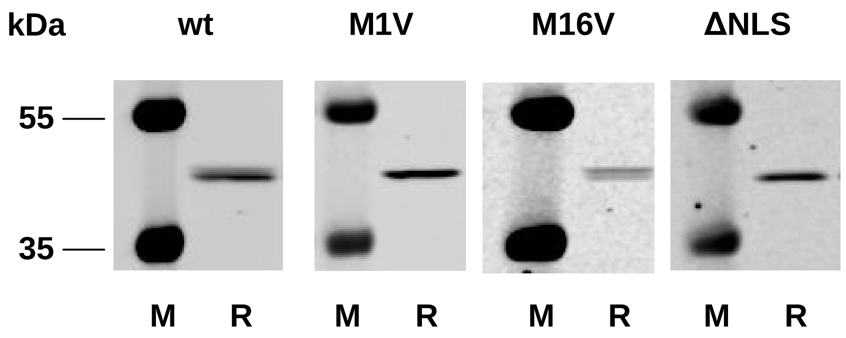
kDa
wt
M1V
M16V
ΔNLS
55
35
M R
M R
M R
M R

F

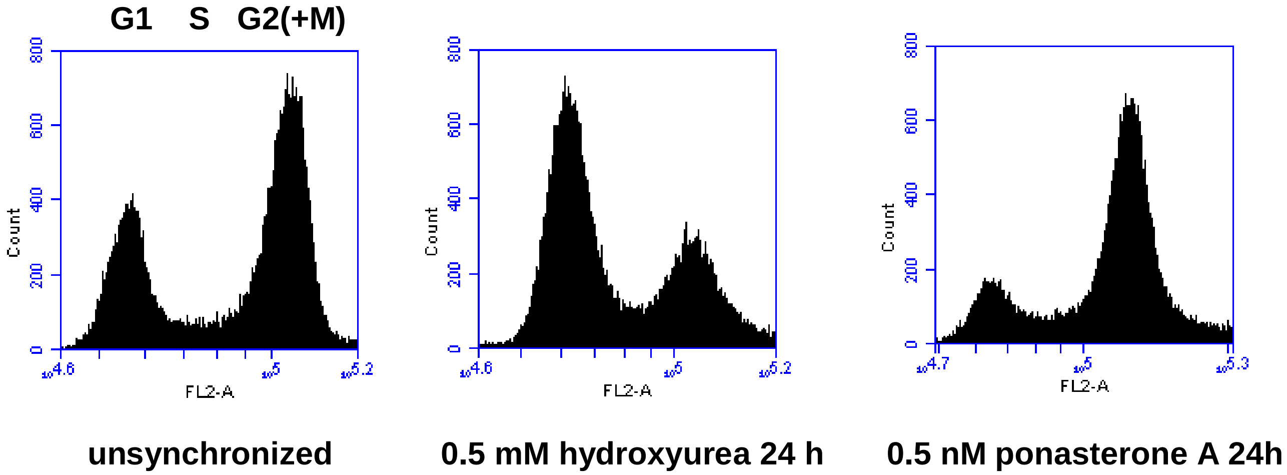
G1 S G2(+M)
Count
FL2-A
unsynchronized
0.5 mM hydroxyurea 24 h
0.5 nM ponasterone A 24h

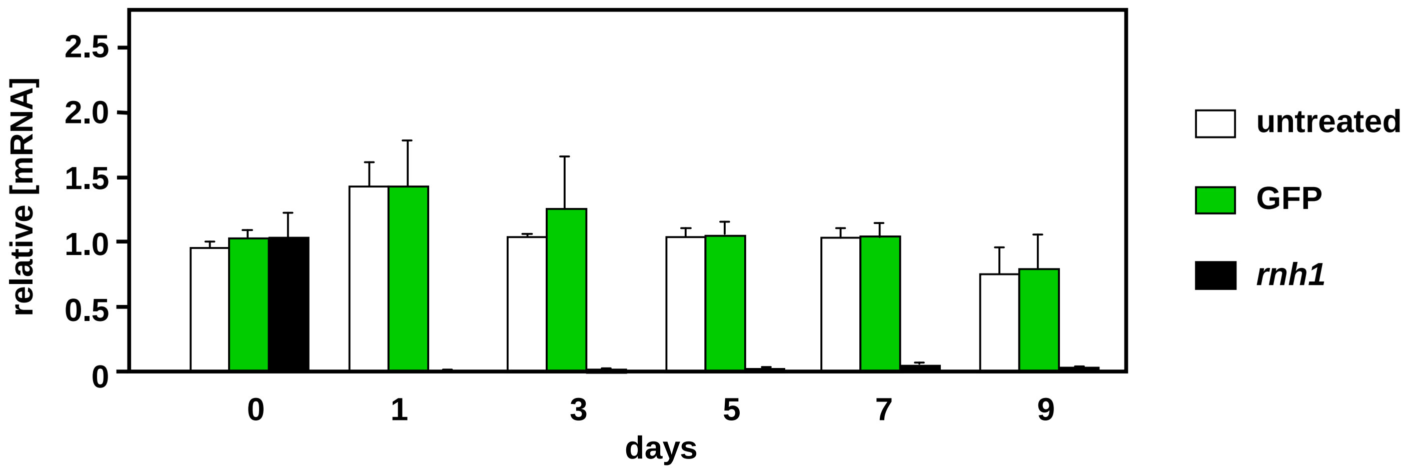

González de Cózar et al, Figure S3

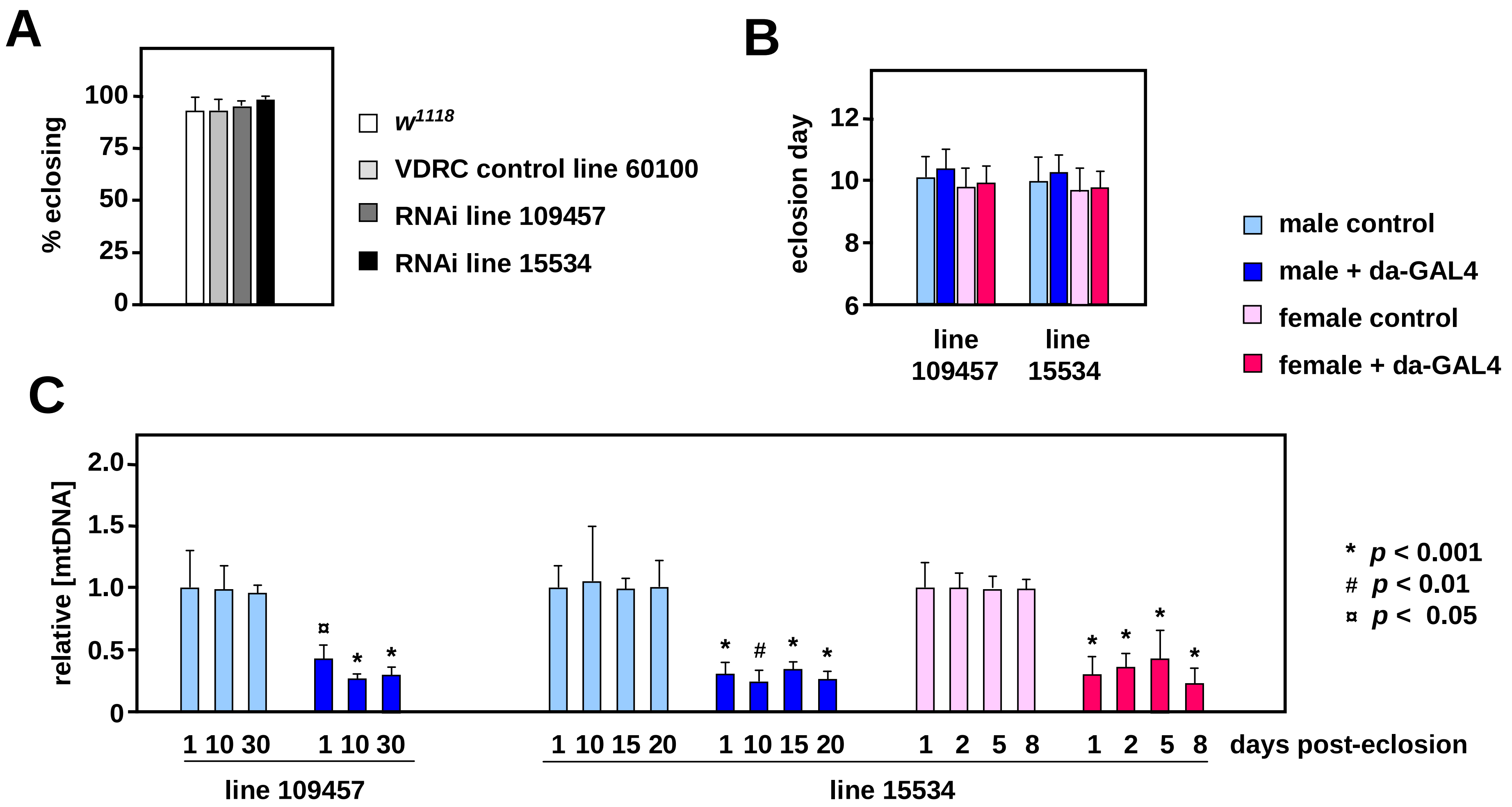

González de Cózar et al, Figure S4

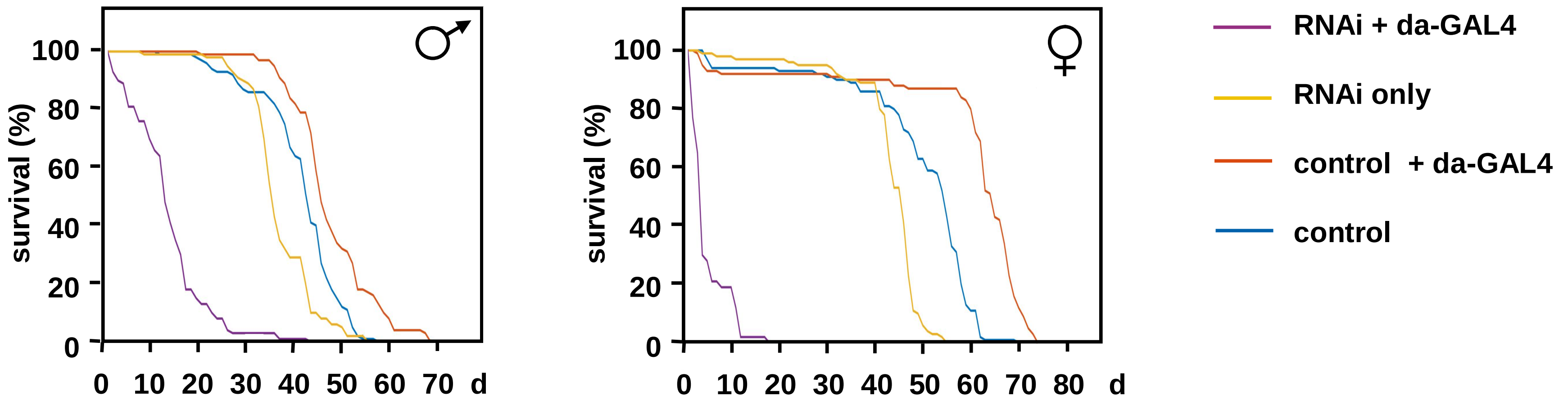

González de Cózar et al, Figure S5

A

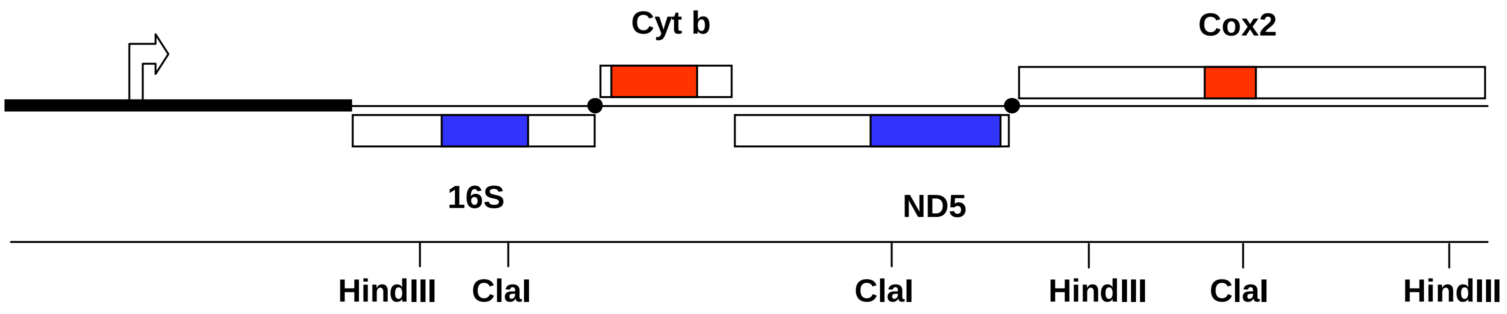

B

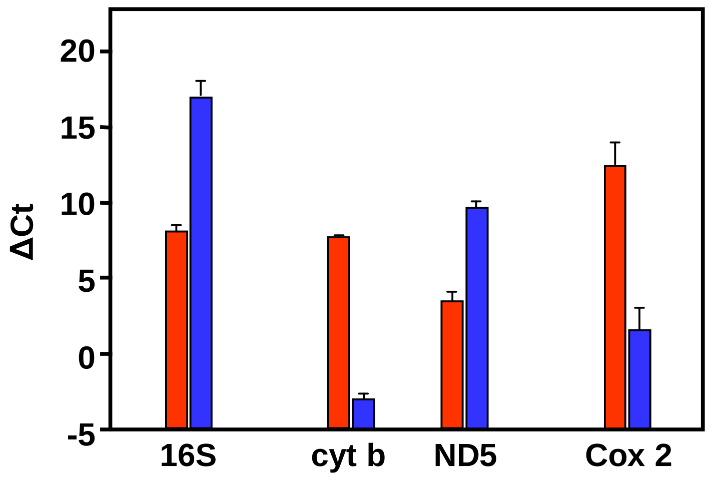

González de Cózar et al, Figure S6

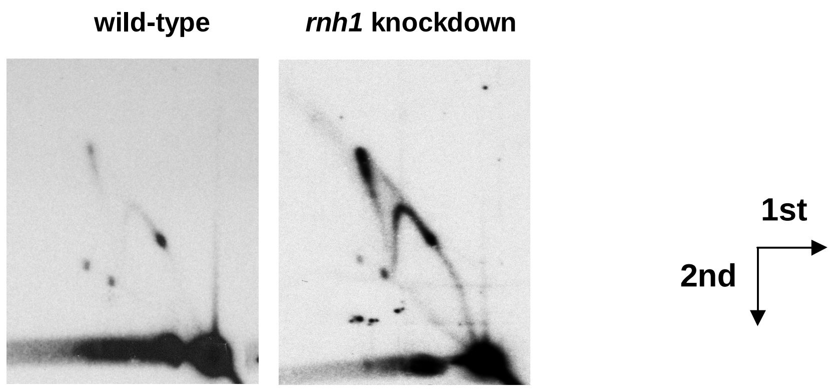

ClaI, probe 6, 4.6 kb

González de Cózar et al, Figure S7

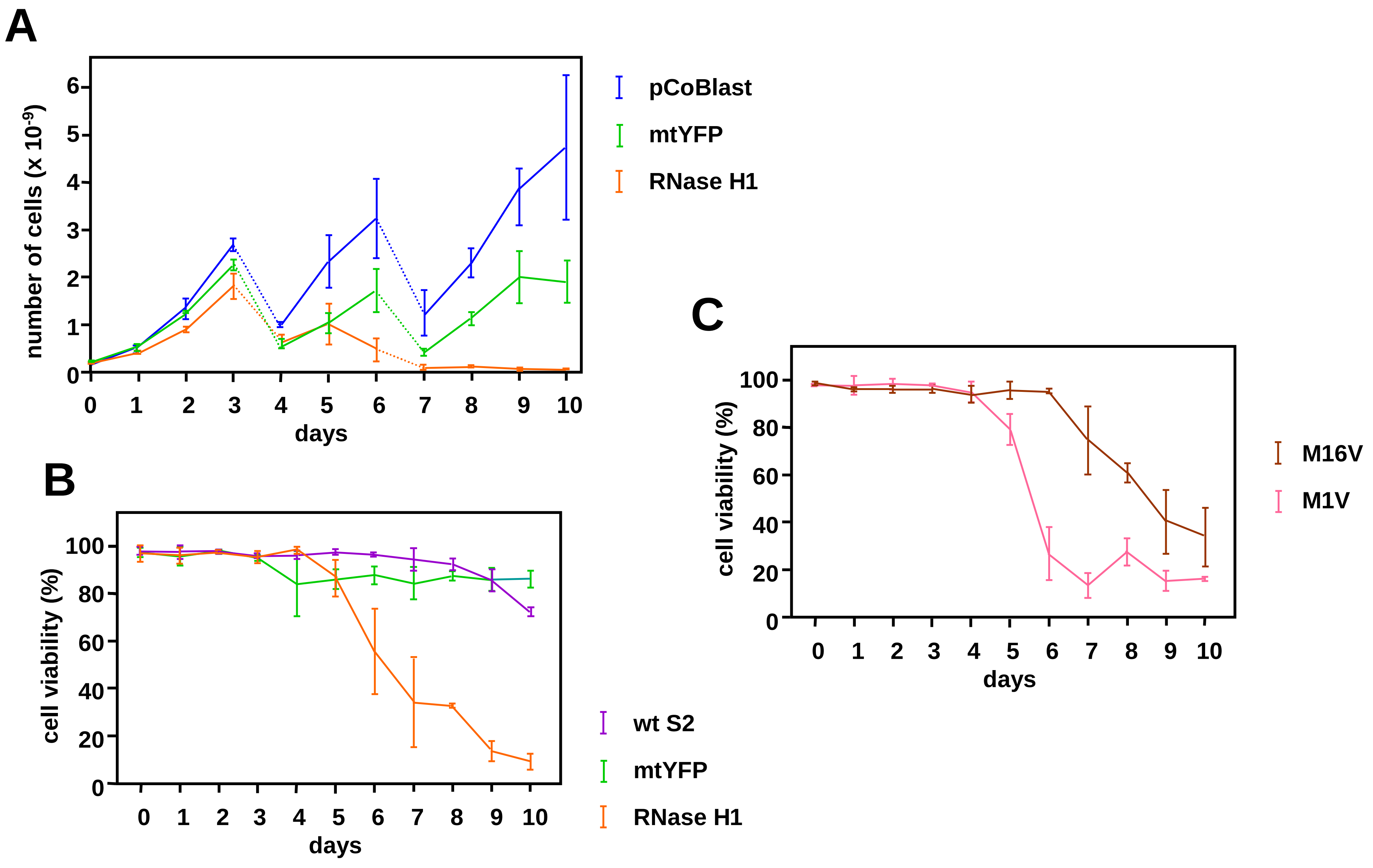

González de Cózar et al, Figure S8
